# Addition of docetaxel, zoledronic acid, or both to first-line long-term hormone therapy in prostate cancer (STAMPEDE): survival results from an adaptive, multiarm, multistage, platform randomised controlled trial

**DOI:** 10.1016/S0140-6736(15)01037-5

**Published:** 2016-03-19

**Authors:** Nicholas D James, Matthew R Sydes, Noel W Clarke, Malcolm D Mason, David P Dearnaley, Melissa R Spears, Alastair W S Ritchie, Christopher C Parker, J Martin Russell, Gerhardt Attard, Johann de Bono, William Cross, Rob J Jones, George Thalmann, Claire Amos, David Matheson, Robin Millman, Mymoona Alzouebi, Sharon Beesley, Alison J Birtle, Susannah Brock, Richard Cathomas, Prabir Chakraborti, Simon Chowdhury, Audrey Cook, Tony Elliott, Joanna Gale, Stephanie Gibbs, John D Graham, John Hetherington, Robert Hughes, Robert Laing, Fiona McKinna, Duncan B McLaren, Joe M O'Sullivan, Omi Parikh, Clive Peedell, Andrew Protheroe, Angus J Robinson, Narayanan Srihari, Rajaguru Srinivasan, John Staffurth, Santhanam Sundar, Shaun Tolan, David Tsang, John Wagstaff, Mahesh K B Parmar

**Affiliations:** a Warwick Medical School, University of Warwick, Coventry, UK; b University Hospitals Birmingham NHS Foundation Trust, The Medical School, University of Birmingham, Birmingham, UK; c MRC Clinical Trials Unit at UCL, London, UK; d Department of Urology, The Christie and Salford Royal NHS Foundation Trusts, Manchester, UK; e Cardiff University School of Medicine, Velindre Hospital, Cardiff, UK; f The Institute of Cancer Research & Royal Marsden NHS Foundation Trust, London, UK; g Institute of Cancer Sciences, University of Glasgow, Beatson West of Scotland Cancer Centre, Glasgow, UK; h Department of Urology, Leeds Teaching Hospitals NHS Trust, Leeds; i Department of Urology, University Hospital, Bern, Switzerland; j Patient rep, MRC Clinical Trials Unit at UCL, London, UK; k Department of Oncology, Weston Park Hospital, Sheffield & Doncaster, UK; l Kent Oncology Centre, Maidstone Hospital, Maidstone, UK; m Department of Oncology, Rosemere Cancer Centre, Royal Preston Hospital, Preston, UK; n Department of Oncology, Poole Hospital NHS Foundation Trust and Royal Bournemouth Hospital NHS Foundation Trust, Chur, Switzerland; o Kantonsspital Graubünden, Chur, Switzerland; p Department of Oncology, Derby Hospitals NHS Foundation Trust, Royal Derby Hospital, Derby, UK; q Department of Medical Oncology, Guy's Hospital, London, UK; r Department of Oncology, Cheltenham General Hospital & Hereford County Hospital, UK; s Department of Clinical Oncology, The Christie NHS Foundation Trust, Manchester, UK; t Oncology and Haematology Clinical Trials Unit, Queen Alexandra Hospital, Portsmouth, UK; u Department of Oncology, Queen's Hospital, Romford, UK; v Beacon Centre, Musgrove Park Hospital, Taunton, UK; w Department of Urology, Hull & East Yorkshire Hospitals NHS Trust, Hull, UK; x Mount Vernon Group, Mount Vernon Hospital, Middlesex, UK; y Department of Oncology, Royal Surrey County Hospital, Guildford, UK; z Department of Oncology, East Sussex Hospitals Trust, East Sussex, UK; aa Department of Oncology, Western General Hospital, Edinburgh, UK; ab Centre for Cancer Research and Cell Biology, Queens University Belfast/Belfast City Hospital, Belfast, UK; ac Department of Oncology, East Lancashire Hospitals NHS Trust, East Lancashire, UK; ad Department of Oncology & Radiotherapy, South Tees NHS Trust, Middlesbrough, UK; ae Department of Oncology, Churchill Hospital, Oxford, UK; af Department of Oncology, Sussex Cancer Centre, Brighton, UK; ag Department of Oncology, Shrewsbury & Telford Hospitals NHS Trust, Shrewsbury, UK; ah Department of Oncology, Royal Devon & Exeter Hospital, Exeter, UK/Torbay Hospital, Torquay, UK; ai Department of Oncology, Nottingham University Hospitals NHS trust, Nottingham, UK; aj Department of Oncology & Radiotherapy, Clatterbridge Cancer Centre, Wirral, UK; ak Department of Oncology, Southend & Basildon Hospitals, Essex, UK; al The South West Wales Cancer Institute and Swansea University College of Medicine, Swansea, UK

## Abstract

**Background:**

Long-term hormone therapy has been the standard of care for advanced prostate cancer since the 1940s. STAMPEDE is a randomised controlled trial using a multiarm, multistage platform design. It recruits men with high-risk, locally advanced, metastatic or recurrent prostate cancer who are starting first-line long-term hormone therapy. We report primary survival results for three research comparisons testing the addition of zoledronic acid, docetaxel, or their combination to standard of care versus standard of care alone.

**Methods:**

Standard of care was hormone therapy for at least 2 years; radiotherapy was encouraged for men with N0M0 disease to November, 2011, then mandated; radiotherapy was optional for men with node-positive non-metastatic (N+M0) disease. Stratified randomisation (via minimisation) allocated men 2:1:1:1 to standard of care only (SOC-only; control), standard of care plus zoledronic acid (SOC + ZA), standard of care plus docetaxel (SOC + Doc), or standard of care with both zoledronic acid and docetaxel (SOC + ZA + Doc). Zoledronic acid (4 mg) was given for six 3-weekly cycles, then 4-weekly until 2 years, and docetaxel (75 mg/m^2^) for six 3-weekly cycles with prednisolone 10 mg daily. There was no blinding to treatment allocation. The primary outcome measure was overall survival. Pairwise comparisons of research versus control had 90% power at 2·5% one-sided α for hazard ratio (HR) 0·75, requiring roughly 400 control arm deaths. Statistical analyses were undertaken with standard log-rank-type methods for time-to-event data, with hazard ratios (HRs) and 95% CIs derived from adjusted Cox models. This trial is registered at ClinicalTrials.gov (NCT00268476) and ControlledTrials.com (ISRCTN78818544).

**Findings:**

2962 men were randomly assigned to four groups between Oct 5, 2005, and March 31, 2013. Median age was 65 years (IQR 60–71). 1817 (61%) men had M+ disease, 448 (15%) had N+/X M0, and 697 (24%) had N0M0. 165 (6%) men were previously treated with local therapy, and median prostate-specific antigen was 65 ng/mL (IQR 23–184). Median follow-up was 43 months (IQR 30–60). There were 415 deaths in the control group (347 [84%] prostate cancer). Median overall survival was 71 months (IQR 32 to not reached) for SOC-only, not reached (32 to not reached) for SOC + ZA (HR 0·94, 95% CI 0·79–1·11; p=0·450), 81 months (41 to not reached) for SOC + Doc (0·78, 0·66–0·93; p=0·006), and 76 months (39 to not reached) for SOC + ZA + Doc (0·82, 0·69–0·97; p=0·022). There was no evidence of heterogeneity in treatment effect (for any of the treatments) across prespecified subsets. Grade 3–5 adverse events were reported for 399 (32%) patients receiving SOC, 197 (32%) receiving SOC + ZA, 288 (52%) receiving SOC + Doc, and 269 (52%) receiving SOC + ZA + Doc.

**Interpretation:**

Zoledronic acid showed no evidence of survival improvement and should not be part of standard of care for this population. Docetaxel chemotherapy, given at the time of long-term hormone therapy initiation, showed evidence of improved survival accompanied by an increase in adverse events. Docetaxel treatment should become part of standard of care for adequately fit men commencing long-term hormone therapy.

**Funding:**

Cancer Research UK, Medical Research Council, Novartis, Sanofi-Aventis, Pfizer, Janssen, Astellas, NIHR Clinical Research Network, Swiss Group for Clinical Cancer Research.

**Research in Context**

**Evidence before this study**

Systemic treatment has changed little for newly diagnosed men with high-risk locally advanced or metastatic prostate cancer since the development of gonadotropin-releasing hormone analogues a generation ago. The only major change has been the use of radical radiotherapy for men whose disease had not spread. This century, new agents began to show valuable activity in relapsed, metastatic, castrate-refractory prostate cancer—including zoledronic acid, which was approved in 2002 on the basis of reduced morbidity in men with bone metastases (a site of spread in up to 90% of castrate-refractory prostate cancer), and docetaxel, with improved survival demonstrated in 2004. Several other trials in addition to STAMPEDE have assessed both drugs in the newly diagnosed setting, most notably GETUG-15 and CHAARTED, which assessed docetaxel in the metastatic setting (about 60% of the population used in our trial). A systematic review and meta-analysis was done in parallel to preparation of this report and contains details of the review strategy.

**Added value of this study**

Our results for zoledronic acid show no convincing evidence of worthwhile benefit either on failure-free or overall survival. These results are congruent with emerging results from other trials in men starting long-term hormone therapy. The docetaxel results showed an improvement in overall survival (HR 0·78; 95% CI 0·66–0·93; p=0·006). There was a notable improvement in survival for the metastatic subset, which is consistent with findings from GETUG-15 and CHAARTED which both also showed gains in failure-free survival with docetaxel. GETUG-15 showed a non-significant improvement in overall survival, and CHAARTED reported a statistically significant improvement in overall survival.

**Implications of all the available evidence**

Together, these trials provide evidence that six cycles of docetaxel should be added to standard androgen deprivation therapy for men with metastatic disease commencing treatment. Men with non-metastatic disease had better prognoses, and failure-free survival was clearly improved by docetaxel; however, there were relatively few deaths in those with non-metastatic disease, so statements about overall survival in this population remain underpowered.

## Introduction

Since October, 2005, the STAMPEDE randomised controlled trial has recruited men with metastatic (M1), high-risk localised (N0), or node-positive (N+) prostate cancer who were newly diagnosed or had high-risk recurrent disease following previous local therapy. All were commencing first-line long-term hormone therapy. Prognosis for these patient groups had altered little since the first description of the effects of hormone therapy in the 1940s. This began to change in the early 21st century with licensing of agents that improved survival (ie, docetaxel, abiraterone, enzalutamide, cabazitaxel, radium-223, and sipuleucel-T),^1^, ^2^, ^3^, ^4^, ^5^, ^6^, ^7^, ^8^ and disease-modifying agents that reduced morbidity (zoledronic acid and denosumab).^9^, ^10^ However, these agents have all shown their benefits in the setting of castrate-refractory prostate cancer (ie, after first-line hormone therapy has ceased working).

STAMPEDE uses a multiarm, multistage (MAMS) platform design to test whether the addition of treatments at the time of long-term hormone therapy initiation improves overall survival. Here, we evaluate and report findings for zoledronic acid and docetaxel; data for celecoxib, abiraterone, enzalutamide in combination with abiraterone, and (in patients with newly diagnosed metastatic disease only) prostate radiotherapy will be reported elsewhere. We have previously reported the celecoxib-containing groups which closed to recruitment early after a pre-planned second intermediate analysis failed to show sufficient effect on failure-free survival.^11^ We have also previously reported control group outcome data for patients with metastatic^12^ and non-metastatic^13^ disease. We report here the first survival data for the following original groups in this platform trial: zoledronic acid, docetaxel, and their combination. Other trials have also examined similar strategies, both in patients with non-metastatic disease and in those with metastatic disease, usually with single drugs. A meta-analysis with other docetaxel and zoledronic acid-containing trials has been conducted and is reported separately.^14^

Zoledronic acid was licensed in 2002 on the basis of an improvement in a composite outcome measure of time to first skeletal-related event, with a risk ratio of 0·64 (95% CI 0·49 to 0·85) in castrate-refractory prostate cancer, and subsequent reduction in further skeletal-related events using the 4 mg schedule.^9^ There was limited evidence of a benefit in survival, although the study was underpowered. Two previous UK trials, PR04 and PR05, used sodium clodronate in patients with non-metastatic and metastatic disease, respectively. The PR05 trial showed improved survival with concurrent hormone therapy plus clodronate but no evidence of benefit was seen in PR04.^15^

Docetaxel (75 mg/m^2^) 3-weekly (ie, given every 3 weeks) was licensed for metastatic castrate-refractory prostate cancer in 2004, on the basis of two trials comparing mitoxantrone and prednisone with docetaxel and either prednisone^2^ or estramustine.^1^ The median survival benefit observed was about 3 months, with a hazard ratio (HR) of 0·76 (0·62–0·94)^2^ for docetaxel compared with mitoxantrone.

The STAMPEDE trial^16^, ^17^ used interim activity analyses, based on failure-free survival, to select groups to continue accrual for fully powered survival analysis. We report here overall, failure-free, and prostate-cancer-specific survival data from the zoledronic acid and docetaxel groups and their combination, together with adverse event data and treatment after relapse.

## Methods

### Study design and participants

We used a MAMS platform trial approach, incorporating a seamless phase 2/3 design.^18^ The rationale and design have been described previously.^16^, ^17^, ^19^ Full details are in the protocol. In summary, eligible patients had prostate cancer that was newly diagnosed as metastatic, node positive, or high-risk locally advanced (with at least two of T3/4, Gleason score of 8–10, and prostate-specific antigen ≥40 ng/mL); or previously treated with radical surgery, radiotherapy, or both and relapsing with high-risk features. All patients were intended for long-term hormone therapy, started no longer than 12 weeks before randomisation. There were no age restrictions; patients were required to be fit for chemotherapy with no clinically significant cardiovascular history.

### Randomisation and masking

Patients were randomised centrally using a computerised algorithm, developed and maintained by the trials unit. Minimisation with a random element of 80% was used, stratifying for hospital, age at randomisation, presence of metastases, planned radiotherapy use, nodal involvement, WHO performance status, planned hormone therapy, and regular use of aspirin or another non-steroidal anti-inflammatory drug. Allocation was in a 2:1:1:1 ratio to standard of care only (SOC-only), standard of care plus zoledronic acid (SOC + ZA), standard of care plus docetaxel (SOC + Doc), or standard of care plus zoledronic acid and docetaxel (SOC + ZA + Doc). Masking to treatment allocation was considered impracticable and of limited value given the primary outcome measure.

### Procedures

Standard of care was hormone therapy for at least 2 years with gonadotropin-releasing hormone agonists or antagonists or, only between 2006 and 2011 for patients with non-metastatic disease, oral anti-androgens alone. Orchidectomy was an allowable alternative to drug therapy. No recommendations around the use of granulocyte colony stimulating factor with docetaxel were given. Radiotherapy, at 6–9 months after randomisation, was encouraged for patients with N0M0 disease, until November, 2011, then mandated; radiotherapy was optional for patients with N+M0 disease; staging was with the Union for International Cancer Control (UICC) TNM staging criteria. Guidance on radiotherapy techniques are described elsewhere.^13^ Zoledronic acid (4 mg) was given for six 3-weekly cycles, then 4-weekly until 2 years. Docetaxel (75 mg/m^2^) was given for six 3-weekly cycles with prednisolone (10 mg) daily, and standard premedication before each injection. Dose modifications were described in the protocol. Trial therapy was discontinued after disease progression or intolerable adverse events.

Patients were followed up 6-weekly to 6 months, 12-weekly to 2 years, 6-monthly to 5 years, then annually. Prostate-specific antigen was measured at every follow-up; further tests were at the clinician's discretion. Nadir prostate-specific antigen was the lowest value reported within 24 weeks after enrolment. Adverse events were graded with Common Toxicity Criteria (CTCAE) version 3.0; toxic effects and symptoms were reported at regular follow-up visits. Serious adverse events, including serious adverse reactions, were reported accordingly. The trial was done in accordance with Good Clinical Practice guidelines and the Declaration of Helsinki, and had the relevant regulatory and ethics approvals (eg, in the UK we obtained national ethics approval, national regulatory approval, and local implementation). All patients gave written, informed consent.

### Outcomes

The definitive and intermediate primary outcome measures were overall survival and failure-free survival, respectively. Overall survival was defined as time from randomisation to death from any cause. Failure-free survival, which is commonly used to drive decisions in the clinic, was selected because it is on the causal pathway to death from prostate cancer and was not required to be a surrogate for overall survival. It was defined as time from randomisation to first evidence of at least one of: biochemical failure; progression either locally, in lymph nodes, or in distant metastases; or death from prostate cancer. Biochemical failure was defined as a rise of 50% above the within-24-week nadir and above 4 ng/mL and confirmed by retest or treatment.^12^ We expected prostate-specific antigen failure to be the most common manifestation of failure-free survival events. Cause of death was determined by masked central review. Death from prostate cancer was recorded when classified by the reviewer as “definitely” or “probably” prostate cancer. The site investigator's determination was used for deaths not yet reviewed.

### Statistical analysis

The sample size was calculated using nstage and its predecessor programmes in Stata, which enables design of MAMS trials.^20^ Assuming, for the control group, 2 years' median failure-free survival, and median overall survival between 4 and 5 years, we targeted a 25% relative improvement (HR 0·75) in both failure-free survival and overall survival for each comparison of research group with control. Accumulating data were reviewed by an Independent Data Monitoring Committee, guided by lack-of-benefit stopping guidelines.^16^, ^17^, ^18^ The efficacy stage analysis of each pairwise comparison of research against control for overall survival required around 400 deaths in the control arm for 90% power and 2·5% one-sided α (corresponding to a two-sided α of 5%), accounting for three intermediate analyses on failure-free survival (analysed March, 2010, April, 2011, and May, 2012). The research groups within STAMPEDE were seen to test distinct hypotheses, and the trial was purposely not designed as a factorial trial.^17^ In this situation, many methodologists would not be concerned about the family-wise error rate.^3^, ^21^, ^22^, ^23^ However, for completeness we calculated the maximum family-wise error rate as 6·75% for these three research groups.

Patients without the event of interest were censored at the time last known to be event free. Standard survival analysis methods were used to analyse time-to-event data. Cox proportional hazards regression models were used to estimate most relative treatment effects. This model was adjusted for stratification factors (except hospital and planned hormone therapy), and stratified by time periods defined by addition of a new research group or end in recruitment to an ongoing research group. An HR below 1·00 favoured the research group. Flexible parametric models were constructed with 4 degrees of freedom for each of the baseline hazard function and time-dependent effect, and adjusted for stratification factors and time periods.^24^ Medians and 5-year estimates come from the flexible parametric model fitted to the data; these are more reliable than reading the Kaplan-Meier curves. The proportional hazards assumption was tested; restricted mean survival time was emphasised in the presence of non-proportionality. Fine and Gray regression models were used for competing risk analysis of prostate-cancer-specific survival (non-metastatic prostate-cancer-specific survival analyses did not adjust for time period due to lack of convergence). Prespecified analyses looked at consistency of treatment effect within stratification factors, over time period, and also by categorised Gleason score (≤7, 8+, unknown), recurrent disease, and prostate-specific antigen values before hormone therapy. The statistical analysis plan was modified to include an analysis of the subset of patients with metastatic disease at randomisation after the presentation of CHAARTED^25^ and GETUG-15^26^ and before this primary analysis was performed. All tests were two-sided, with confidence intervals given at the 95% level.

Median follow-up was determined through the standard approach of reverse-censoring on death, in which survival is treated as the event and death as censoring. All patients are included in the efficacy analyses according to allocated treatment on an intention-to-treat basis. Adverse event data are shown for the safety population, comprising patients who received any study drug and underwent adverse event assessment, and analysed according to treatment initiated irrespective of study group assignment; a sensitivity analysis of safety was done on an intention-to-treat basis. Safety data were assessed continuously; we also present a safety analysis at 1 year, chosen to assess whether chemotherapy side-effects had ameliorated by this timepoint. A formal comparison of those research groups showing a survival advantage, compared with SOC-only, was done, and a pre-planned factorial analysis (without an interaction term) is included for completeness. Data on first skeletal-related event and osteonecrosis of the jaw are also presented. All other analyses are exploratory. Statistical analyses were done with Stata version 14 and nstage. The trial is registered at ClinicalTrials.gov (NCT00268476) and ControlledTrials.com (ISRCTN78818544).

### Role of the funding source

The trial was sponsored by the MRC and conducted by the MRC Clinical Trials Unit at UCL with the Swiss Group for Clinical Cancer Research. MRC employees were central to the conduct of the trial and the development of this manuscript. Only authors MRSp and MRSy had access to raw data; processed data released by the Independent Data Monitoring Committee and Trial Steering Committee were available to all coauthors. Cancer Research UK (MRC PR08, CRUK/06/019) approved, but had no further input into, the trial design. Pfizer, Novartis, and Sanofi-Aventis approved the initial and amended trial design and participated in discussions on the progress of the trial. Representatives from these industry partners were invited to comment on the report. The analyses were driven by prespecified criteria and the decision to submit for publication was made by the Trial Management Group.

## Results

**Figure 1:**
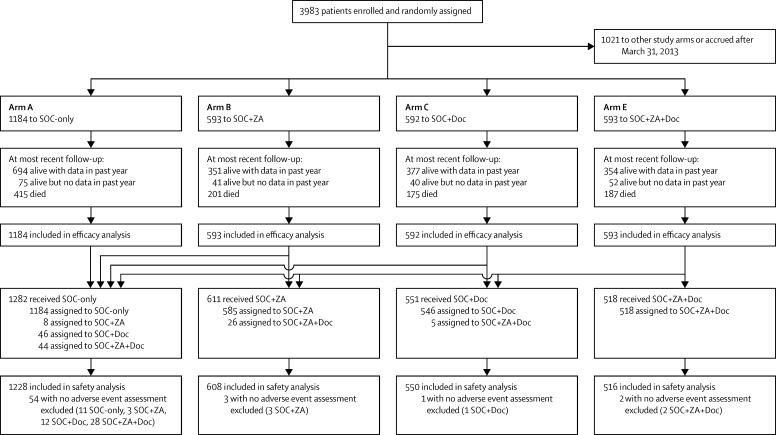
Trial profile SOC-only=standard of care only. SOC + ZA=standard of care plus zoledronic acid. SOC + Doc=standard of care plus docetaxel. SOC + ZA + Doc=standard of care plus zoledronic acid and docetaxel.

Between Oct 5, 2005, and March 31, 2013, 2962 patients were randomised from more than 100 UK and Swiss sites: 1184 to SOC-only, 593 SOC + ZA, 592 SOC + Doc, and 593 SOC + ZA + Doc. Data were frozen on May 13, 2015, with a cutoff of March 4, 2015. The appendix shows an overview of the broader trial design and groups recruiting over time, whereas figure 1 shows the CONSORT flow diagram for analyses presented here. Table 1 gives baseline characteristics of these patients. Median follow-up was 43 months (IQR 30–60). Most patients (94%) were newly diagnosed. 1738 (62%) of 2797 newly diagnosed patients had metastatic disease at entry, compared with 79 (48%) of 165 patients with recurrent disease. Median age was 65 years (IQR 60–71), median prostate-specific antigen 65 ng/mL (IQR 23–184), and 2092 (71%) patients were Gleason score 8–10.

Median time to starting zoledronic acid was about 2 weeks after randomisation, and about 8 weeks from starting hormone therapy (most patients started hormone therapy before randomisation). Median duration of zoledronic acid was 16·6 months (IQR 7·8–23·2) for SOC + ZA and 19·5 months (IQR 9·1–23·4) for SOC + ZA + Doc, with the difference in duration being driven by differences in time to progression (table 2). Of patients allocated to receive zoledronic acid as part of trial treatment, overall about 40% of patients completed 2 years of zoledronic acid therapy (table 2). When less than 2 years of treatment was received, progression was the most common reason for stopping. Zoledronic acid was not started in eight (1%) patients assigned to SOC + ZA and 49 (8%) patients assigned to SOC + ZA + Doc, mostly due to treatment refusal.

Median time to starting docetaxel was about 2 weeks after randomisation and 9 weeks after starting hormone therapy. Of patients allocated to receive docetaxel as part of trial treatment, 456 (77%) patients assigned to SOC + Doc and 422 (71%) to SOC + ZA + Doc received the full six cycles, whereas 477 (81%) assigned to SOC + Doc and 446 (75%) to SOC + ZA + Doc received at least five cycles (table 3). When five or fewer cycles were reported, toxic effects were the most common reason for stopping (table 3), with few patients reporting stopping for disease progression. Docetaxel was not started in 46 (8%) patients assigned to SOC + Doc and 70 (12%) to SOC + ZA + Doc, again mostly due to treatment refusal, patient choice, or withdrawal from the trial.

Planned use of standard of care radiotherapy was similar across groups (28–29%), with reported use being 323 (27%) patients for SOC-only; 155 (26%) for SOC + ZA; 154 (26%) for SOC + Doc; and 148 (25%) for SOC + ZA + Doc. In patients with non-metastatic disease, 62% were planned for radiotherapy, with the corresponding figures for reported use being 289 (63%) for SOC-only, 136 (60%) for SOC + ZA, 131 (57%) for SOC + Doc, and 130 (57%) for SOC + ZA + Doc; higher proportions of N0 than N+ patients received radiotherapy (appendix page 9).

There were 415 deaths (347 prostate cancer deaths; 84%) in patients receiving SOC-only; median survival was 71 months (IQR 32 to not reached) and 5-year survival was 55%. These data form the reference for each comparison of research group with control.

**Figure 2:**
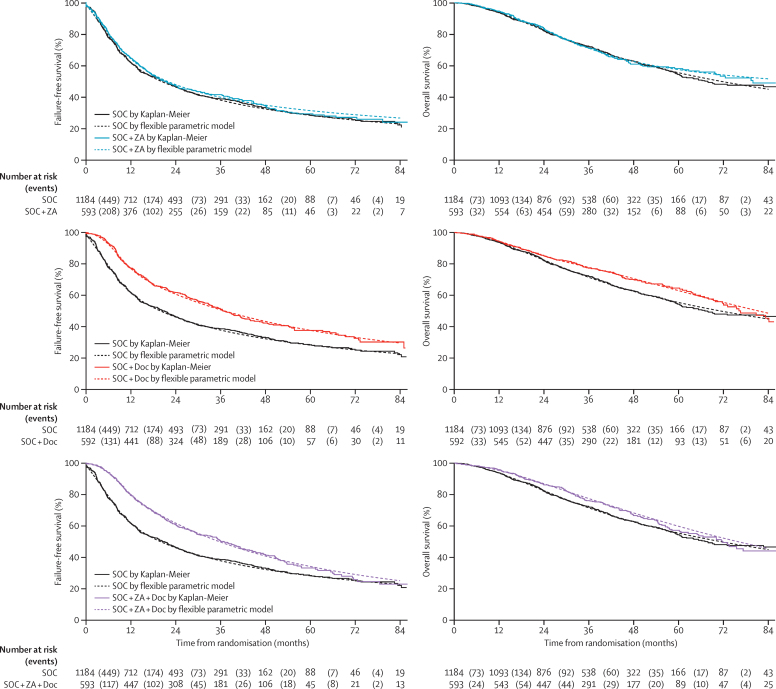
Failure-free and overall survival Figure shows Kaplan-Meier curves and flexible parametric models fitted to the data. Number at risk (events) shows the number of individuals at risk (ie, the number who were event free) at each timepoint, with parentheses showing the number of individuals who developed events in the period between each timepoint. SOC-only=standard of care only. SOC + ZA=standard of care plus zoledronic acid. SOC + Doc=standard of care plus docetaxel. SOC + ZA + Doc=standard of care plus zoledronic acid and docetaxel.

201 patients in the SOC + ZA group died (169 prostate cancer; 84%), with no evidence of a survival advantage compared with SOC-only (HR 0·94, 95% CI 0·79–1·11; p=0·450); median survival was not reached (IQR 32 to not reached), and 5-year survival was 57%. However, there was evidence of a survival advantage for SOC + Doc (HR 0·78, 95% CI 0·66–0·93; p=0·006) with 175 deaths (143 prostate cancer; 82%), median survival 81 months (IQR 41 to not reached), and 5-year survival of 63%. There was also evidence of survival advantage for SOC + ZA + Doc (HR 0·82, 95% CI 0·69–0·97; p=0·022) with 187 deaths (150 prostate cancer; 80%), median survival 76 months (IQR 39 to not reached), and 5-year survival of 60%. There was no evidence of non-proportional hazards. Plots for survival are shown in figure 2.

**Figure 3:**
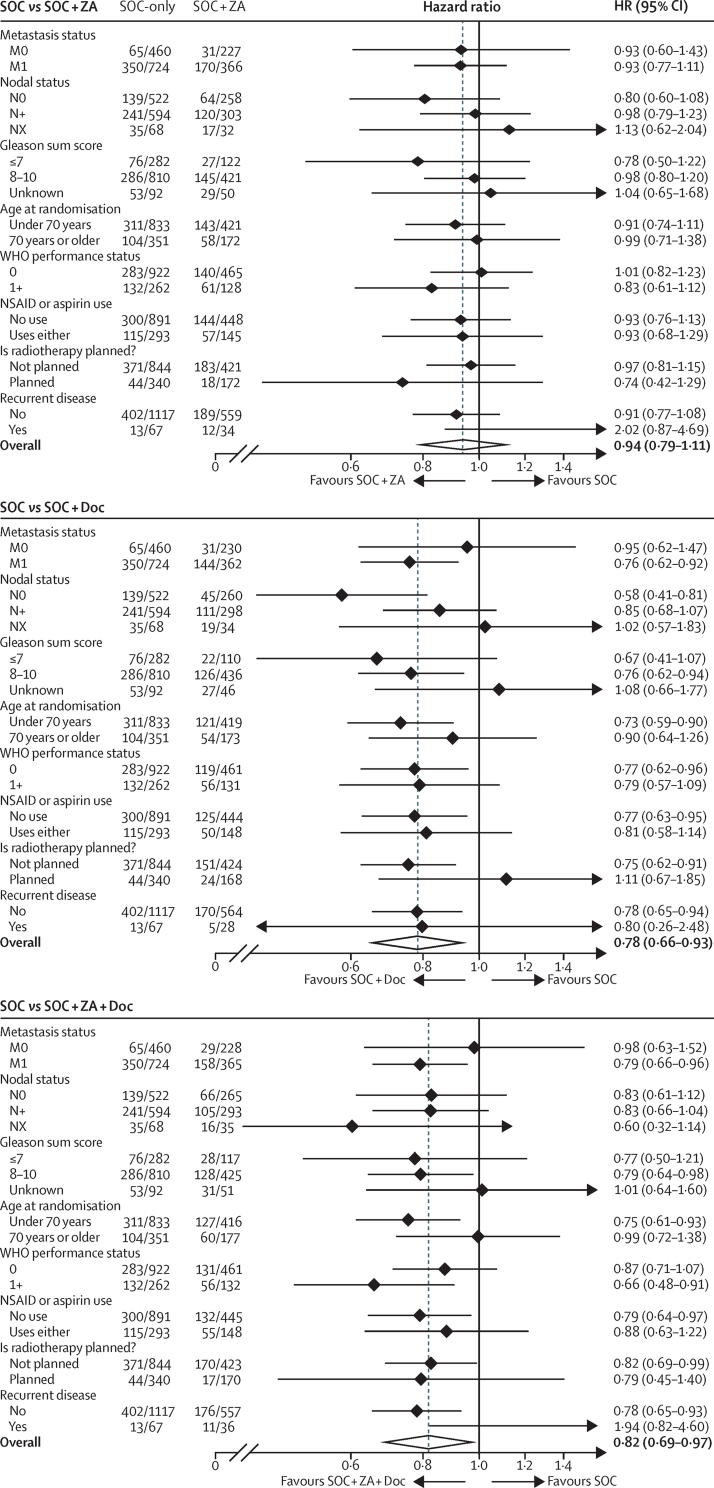
Forest plots of treatment effect on survival within subsets Data are deaths/N or HR (95% CI). All p values were statistically non-significant. For SOC-only *vs* SOC + ZA, all p>0·09, PSA p=0·116, time-period p=1·000. For SOC-only *vs* SOC + Doc, all p>0·06, PSA p=0·589, time-period p=1·000. For SOC-only *vs* SOC + ZA + Doc, all p>0·23 except previously treated p=0·023, PSA p=0·254, time-period p=1·000. X axis provided with natural log scaling. SOC-only=standard of care only. SOC + ZA=standard of care plus zoledronic acid. SOC + Doc=standard of care plus docetaxel. SOC + ZA + Doc=standard of care plus zoledronic acid and docetaxel. PSA=prostate-specific antigen. NSAID=non-steroidal anti-inflammatory drug.

We found no evidence of heterogeneity of treatment effect across predefined subsets (figure 3). Pre-planned subset analyses in all 1817 patients with metastatic disease at randomisation included around 500 deaths per comparison. This included 350 deaths in patients on SOC-only (median survival 45 months [IQR 23–91], 5-year survival 39%). There were 170 deaths on SOC + ZA (HR 0·93, 95% CI 0·77–1·11; p=0·416), with median survival 46 months (IQR 24 to not reached) and 5-year survival of 43%. There were 144 deaths on SOC + Doc (HR 0·76, 95% CI 0·62–0·92; p=0·005), with median survival 60 months (IQR 27–103) and 5-year survival of 50% (appendix page 5). Finally, there were 158 deaths on SOC + ZA + Doc (HR 0·79, 95% CI 0·66–0·96; p=0·015), with median survival 55 months (IQR 29–88) and 5-year survival of 46%. Similar comparisons in patients without metastatic disease at randomisation are immature at this time, with fewer than 100 deaths per comparison.

Comparing the two research groups that demonstrated a survival advantage over the control group (SOC + Doc and SOC + ZA + Doc), we noted no evidence of an advantage when adding zoledronic acid to docetaxel (HR 1·06, 95% CI 0·86–1·30; p=0·592). In an exploratory analysis of the effect of docetaxel on survival in the context of zoledronic acid (ie, comparing SOC + ZA with SOC + ZA + Doc), the hazard ratio was 0·87 (95% CI 0·71–1·06). Analysis of the main effects of zoledronic acid and docetaxel in a single factorial model, without a treatment-interaction term, showed docetaxel to be associated with a survival advantage (HR 0·82, 95% CI 0·72–0·93; p=0·003), but not zoledronic acid (HR 0·98, 95% CI 0·86–1·11; p=0·726). An exploratory factorial model, including an interaction term, found no evidence of treatment interaction (p=0·401); the individual treatment effects were the same as in the pairwise comparisons.

Figure 2 shows the failure-free survival plot for each research comparison, and the appendix page 10 shows the form of that failure-free survival event. There were 761 events in patients on SOC-only; median failure-free survival 20 months; 5-year failure-free survival 28%. With 374 events there was no evidence of improvement in failure-free survival with SOC + ZA (HR 0·92, 95% CI 0·81–1·04; p=0·198); median failure-free survival was 22 months and 5-year failure-free survival was 31%. There was, however, evidence of an improvement in failure-free survival both for SOC + Doc, with 315 events (HR 0·61, 95% CI 0·53–0·70; p=0·413 × 10^−13^), median failure-free survival 37 months, and 5-year failure-free survival 38%; and for SOC + ZA + Doc, with 318 events (HR 0·62, 95% CI 0·54–0·70; p=0·134 × 10^−12^), median failure-free survival 36 months, 5-year failure-free survival 34%. There was strong evidence of non-proportional hazards for both comparisons showing an improvement in failure-free survival (SOC + Doc and SOC + ZA + Doc). In these cases, restricted mean survival time is preferred to the hazard ratio for summarising the treatment effect. Mean failure-free survival, restricted to the first 84 months on trial, was 34·8 months on SOC-only, compared to 44·2 months on SOC + Doc (difference 9·4 months, 95% CI 6·6–12·3; p=0·556 × 10^−10^) and compared to 43·1 months on SOC + ZA + Doc (difference 8·3 months, 95% CI 5·5–11·1; p=0·480 × 10^−8^).

As with survival, there was no evidence of heterogeneity in failure-free survival across the same predefined subsets (appendix page 3). Considering metastatic status subsets, treatment effect was broadly consistent within both non-metastatic and metastatic populations, for all research comparisons, and indicated that docetaxel improved failure-free survival for non-metastatic disease (HR 0·60, 95% CI 0·45–0·80; p=0·283 × 10^−3^) as for metastatic disease (HR 0·61, 95% CI 0·53–0·71; p=0·283 × 10^−10^).

At the time of this analysis, a total of 978 men had died, 809 (83%) from prostate cancer. The proportion of deaths attributed to prostate cancer was increased in men presenting with metastases: 703 (86%) of 822 deaths in the 1817 men presenting with metastases, compared with 106 (68%) of 156 deaths in 1145 men presenting without metastases. Adjusted competing risks regression for prostate-cancer-specific survival showed an advantage over SOC-only for SOC + Doc (subHR 0·79, 95% CI 0·65–0·96; p=0·019) and SOC + ZA + Doc (0·78, 0·65–0·95; p=0·013), but not SOC + ZA (0·95, 0·79–1·15; p=0·613). For patients with metastatic disease, the subHR for SOC + Doc was 0·80 (95% CI 0·65–0·99; p=0·033), for SOC + ZA was 0·92 (0·75–1·12), and for SOC + ZA + Doc was 0·78 (0·64–0·96); for patients with non-metastatic disease, the subHR for SOC + Doc was 0·82 (95% CI 0·48–1·40; p=0·475), for SOC + ZA was 1·08 (0·66–1·76), and for SOC + ZA + Doc was 0·81 (CI 0·46–1·43). We noted particularly limited power for subset analyses at this time for both settings.

Amongst patients randomly assigned to SOC-only, 328 of 1184 reported at least one skeletal-related event. Time to first skeletal-related event was improved with SOC + Doc (112 patients reported skeletal-related event; HR 0·60, 95% CI 0·48–0·74; p=0·127 × 10^−5^) and SOC + ZA + Doc (108 patients; HR 0·55, 95% CI 0·44–0·69; p=0·277 × 10^−7^), but not SOC + ZA (153 patients; HR 0·89, 95% CI 0·73–1·07; p=0·221). There was strong evidence of non-proportional hazards for both comparisons showing improvement in time to first skeletal-related event (SOC + Doc and SOC + ZA + Doc). In these cases, restricted mean survival time is preferred for summarising treatment effect. Mean time to skeletal-related event, restricted to within the first 84 months on trial, was 61·4 months (95% CI 59·5–63·2) on SOC-only, compared with 68·0 months on SOC + Doc (difference 6·6 months, 95% CI 3·6–9·6; p=0·177 × 10^−4^) and 68·3 (65·6–70·3) on SOC + ZA + Doc (difference 6·9 months, 95% CI 4·1–9·8; p=0·249 × 10^−5^). In the patient group with bone metastases at presentation, SOC + ZA similarly had no evidence of an effect (HR 0·94, 95% CI 0·76–1·16; p=0·564).

**Figure 4:**
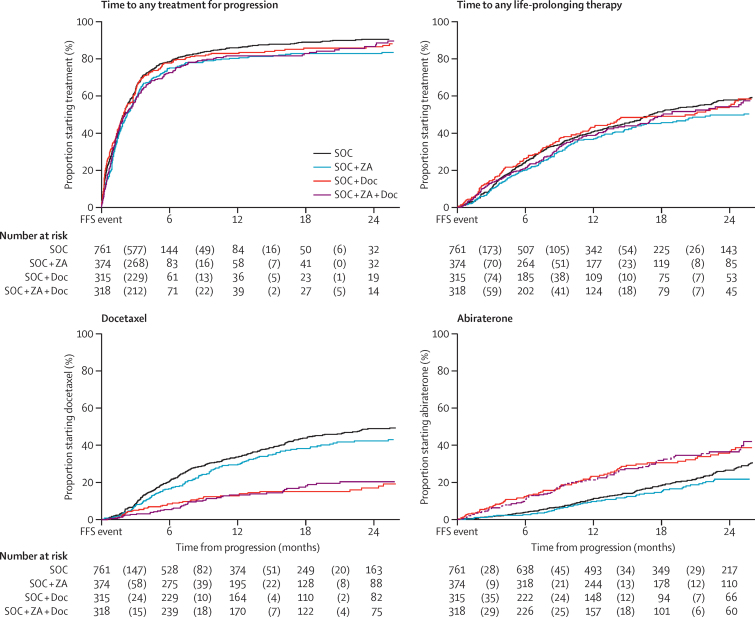
Time to treatment after progression Figure shows treatments ever used at relapse, at the discretion of the treating clinician, by group, cumulative incidence curves. (A) Time to any treatment after progression. (B) Time to any life-prolonging treatment after progression. (C) Time to docetaxel after progression. (D) Time to abiraterone after progression. SOC-only=standard of care only. SOC + ZA=standard of care plus zoledronic acid. SOC + Doc=standard of care plus docetaxel. SOC + ZA + Doc=standard of care plus zoledronic acid and docetaxel. FFS=failure-free survival.

Figure 4 shows time to first of any treatment after a failure-free survival event and time to first life-extending therapy (defined as available agents with proven survival gain in castrate-refractory prostate cancer: docetaxel, abiraterone, cabazitaxel, enzalutamide, and radium-223). There were no obvious differences either in time to any therapy or life-extending therapies between groups. There were however, differences in the pattern of therapy depending on whether patients were docetaxel-exposed upfront (figure 4). Analysis of zoledronic acid use after relapse is provided in the appendix page 7. Overall exposure to treatment for progression is summarised in table 4, showing slightly higher rates of exposure to subsequent therapy in the control group. Use of cabazitaxel, enzalutamide, and radium-223 were low across all groups (appendix page 6).

The proportion of patients reporting worst adverse event ever as grade 3 or higher was highest with SOC + Doc (288 patients [52%]) and SOC + ZA + Doc (269 [52%]; table 5). This was mostly due to events during the first 6 months on trial, when the proportions were 17% (n=203) for SOC-only, 15% (n=91) for SOC + ZA, 36% (n=198) for SOC + Doc, and 39% (n=202) for SOC + ZA + Doc, with docetaxel seeming to contribute the most toxicity. For 1998 patients with adverse event data around 1 year after randomisation (ie, worst adverse event grade reported at 48 or 60 weeks of follow-up), the proportions of grade 3 or higher toxic effects were balanced, with 10% (n=76) patients reporting a worst adverse event as grade 3 or higher with SOC-only, 10% (n=41) with SOC + ZA, 10% with (n=43) SOC + Doc, and 12% (n=49) with SOC + ZA + Doc. The pattern and levels of adverse events were similar in the safety and intention-to-treat populations. There were ten (2%) reported cases of osteonecrosis of the jaw on SOC + ZA and 20 (4%) on SOC + ZA + Doc. There were eight deaths probably or possibly related to the research treatment: one on SOC + Doc (neutropenic sepsis), and seven on SOC + ZA + Doc (four neutropenic sepsis, one pneumocystic pneumonia, one interstitial pneumonitis, and one pneumonia).

## Discussion

The STAMPEDE randomised controlled trial is investigating the effectiveness of the front-line use of various treatments in men commencing long-term hormone therapy for newly diagnosed locally advanced or metastatic prostate cancer, or who have relapsed after local therapy with poor prognosis features. The MAMS design used in STAMPEDE has allowed us to address multiple treatment questions simultaneously within a single trial platform.^18^ We will report further randomised comparisons from STAMPEDE in the coming years (appendix page 2), meaning that, through this single protocol, we will have answered at least eight different primary questions in 15 years. To have addressed as many questions in separate, sequential trials would have taken many decades and far more patients, notably allocated to control groups. We recommend that academic and industry researchers consider this design in the future, to make faster progress and good use of limited trial resources.

These are the first mature, comparative, randomised data to emerge from the trial. We found that the addition of docetaxel to standard of care was associated with improved survival, with an HR of 0·78 and a difference in median survival of 10 months, as well as improvements in prostate-cancer-specific survival, failure-free survival, and skeletal-related events. The combination of zoledronic acid and docetaxel was associated with similar improvements, although the benefit observed was smaller. We will report cost-effectiveness and patient-reported outcomes separately.

Docetaxel is a widely used drug with a familiar toxicity profile. Docetaxel was well tolerated in this population, with most patients completing all six cycles in a timely fashion and good dose intensity. Predictable chemotherapy toxic effects, including neutropenia and febrile neutropenia, were observed but few patients stopped treatment because of side-effects. Toxic effects in both docetaxel-containing groups seemed higher than in previous studies of this drug in patients with castrate-refractory prostate cancer (eg, TAX327), but the studies have used different populations.^27^, ^28^ The protocol made no recommendations about growth factor support, and we have not collected information about its use.^29^, ^30^

Docetaxel significantly prolonged failure-free and overall survival across the trial population with no evidence that the effect varied across different groups in the population; in particular, there is no evidence of a difference of the effect of docetaxel by metastatic status, for either of these outcome measures. The beneficial effect on survival is clear in the larger metastatic subpopulation, which accounted for 61% of patients in the trial and 84% of deaths. There were fewer patients with non-metastatic disease and, with their generally more favourable prognosis, there were relatively few deaths in this group; all survival analyses for this subset are currently underpowered. In this non-metastatic subset of men, death from causes other than prostate cancer was more common than in men with metastatic disease, and therefore any effect of docetaxel on overall survival will be diluted. We will report longer-term follow-up in due course, but note that estimates of the treatment effect in failure-free survival and prostate-cancer-specific survival are extremely similar for patients with and without metastases at presentation.

For zoledronic acid, the results show no evidence of efficacy on failure-free survival, skeletal-related events, or overall survival, despite good compliance with therapy and good levels of exposure, with target duration of 2 years. Few patients stopped treatment for side-effects; the most frequent reason for stopping trial therapy within 2 years was disease progression. This differed between the SOC + ZA and SOC + ZA + Doc groups because failure-free survival was increased in the latter group by docetaxel, indirectly leading to increased exposure to zoledronic acid as well. Despite this increased exposure, zoledronic acid showed no evidence of an advantage when added to docetaxel (HR 1·06).

The effect of docetaxel on survival was positive, but clinically significant toxicity did occur; in clinical practice, consideration could be given to early use of growth factor support to enable treatment delivery. There was one treatment-related death in the SOC + Doc group and seven in the combination group. This difference, combined with a more modest survival benefit for the combination treatment, raises the possibility of some interaction (or antagonism) between docetaxel and zoledronic acid in the treatment of this group of men.

A number of trials have now examined docetaxel in the hormone-naive context in both the non-metastatic and metastatic settings, of which STAMPEDE is the largest.^25^, ^26^, ^31^, ^32^, ^33^, ^34^ These findings are discussed elsewhere but consistently show an improvement in failure-free survival.^14^ The CHAARTED trial^25^ recently reported improved survival in metastatic disease whereas GETUG-15,^26^ a similar trial, did not report a differential effect. Taken with our results, there is compelling evidence that front-line docetaxel substantially improves survival in patients with metastatic disease. In the non-metastatic setting, there are insufficient mature survival results in the literature, so further follow-up and engagement in planned meta-analyses are needed to further delineate the effect of docetaxel on survival in this setting. The impact on failure-free survival is both clear and large in favour of docetaxel in STAMPEDE.

The case mix of patients joining the trial included men with newly diagnosed disease and a small proportion of patients with recurrent disease. The recurrent disease subset is small and thus it is unrealistic to look for statistically reliable results in such men; however, we note that estimates of the effect of docetaxel are consistent with that seen in the population as a whole.

For zoledronic acid, there are now several trials showing no evidence of a survival gain with upfront use,^14^, ^35^, ^36^, ^37^ as discussed in the accompanying meta-analysis. This contrasts with the results from MRC PR05^15^ with sodium clodronate (another bisphosphonate), in which a survival benefit was reported in a metastatic population. The companion non-metastatic trial showed no evidence of an effect on survival with the same agent.^15^

Men in the STAMPEDE trial did better than we had expected in terms of survival. We believe this resulted from second-line and third-line treatments which were unavailable when the trial was designed. The timing of second-line therapy after relapse was similar across groups, but choice of which treatment to use was at the investigator's discretion, and, consequently, was varied. This choice would have been affected by local practice and availability of newer treatments over time, such as abiraterone, enzalutamide, radium-223, and cabazitaxel, as well as allocated treatment in the trial.

There are several strengths to note in the STAMPEDE trial and specifically for the analyses reported here. First, the data were prospectively collected and randomised, from nearly 3000 men with patient characteristics that were well balanced by group, and we achieved good median follow-up (43 months). Second, the data were very recently frozen (May, 2015) at a pre-planned analysis point of roughly 400 control group deaths, meaning the primary outcome results are both well powered and have been reported promptly. Third, the patients contributing to these analyses came from more than 100 sites across the UK and Switzerland, suggesting the results to be generalisable. Fourth, the design allows easy understanding of effect across multiple randomisations. Finally, treatment compliance among patients starting treatment was good.

We are aware that there are also limitations within the data. First, the proportion of patients not starting treatment, especially docetaxel, will have a small diluting effect. Linkage to hospital records is required to report more detailed information on skeletal-related events. The impact of therapies that do not target androgen receptors on recurrence (as assessed by prostate-specific antigen) is likely to be complex. Finally, power for assessing the consistency of effects across subsets is inevitably low; we will report long-term follow-up in due course when the maturity will be much greater both across the trial and particularly in the non-metastatic subset.

In conclusion, we have shown improved survival across a population of men commencing first-line long-term hormone therapy through the addition of docetaxel chemotherapy but not by adding zoledronic acid. Therefore, zoledronic acid should not become part of standard of care. Standard of care should be updated to include docetaxel chemotherapy in suitable patients with metastatic disease, and docetaxel may be considered for men with high-risk non-metastatic prostate cancer with or without radiotherapy.

## Figures and Tables

**Table 1:** Baseline characteristics

		**Standard of care (n=1184)**	**Standard of care plus zoledronic acid (n=593)**	**Standard of care plus docetaxel (n=592)**	**Standard of care plus zoledronic acid and docetaxel (n=593)**
**Age, years**					
Median (IQR)		65 (60–70)	66 (61–71)	65 (61–71)	66 (60–70)
Range		41–82	42–82	40–81	42–84
**Prostate-specific antigen (ng/mL)**					
Median (IQR)		67 (23–200)	59 (22–172)	70 (27–181)	63 (21–183)
Range		0–15747	0–13300	1–9999	1–8503
**Days from diagnosis**					
Median (IQR)		75 (55–99)	76 (56–101)	76 (56–99)	76 (56–100)
Range		0–4070	1–4174	3–5033	6–4485
Missing		5	8	7	6
**Pain from prostate cancer**					
Absent		984 (85%)	496 (84%)	490 (84%)	483 (84%)
Present		179 (15%)	93 (16%)	96 (16%)	94 (16%)
Missing		21	4	6	16
**T category at randomisation**					
T0		7 (1%)	3 (1%)	2 (0%)	2 (0%)
T1		21 (2%)	7 (1%)	0 (0%)	5 (1%)
T2		113 (10%)	53 (9%)	60 (10%)	67 (11%)
T3		756 (64%)	395 (67%)	390 (66%)	371 (63%)
T4		211 (18%)	92 (16%)	105 (18%)	100 (17%)
TX		76 (6%)	43 (7%)	35 (6%)	48 (8%)
**N category at randomisation**					
N0		522 (44%)	258 (44%)	260 (44%)	265 (45%)
N+		594 (50%)	303 (51%)	298 (50%)	293 (49%)
NX		68 (6%)	32 (5%)	34 (6%)	35 (6%)
**Metastases**					
None		460 (39%)	227 (38%)	230 (39%)	228 (38%)
Any metastases		724 (61%)	366 (62%)	362 (61%)	365 (62%)
	Bone metastases	634 (54%)	302 (51%)	307 (52%)	310 (52%)
	Liver metastases	15 (1%)	12 (2%)	6 (1%)	9 (2%)
	Lung metastases	33 (3%)	17 (3%)	13 (2%)	14 (2%)
	Nodal metastases	220 (19%)	120 (20%)	102 (17%)	116 (20%)
	Other metastases	46 (4%)	33 (6%)	25 (4%)	21 (4%)
**Broad disease grouping**					
Newly diagnosed N0M0		256 (22%)	120 (20%)	131 (22%)	131 (22%)
Newly diagnosed N+M0		171 (14%)	88 (15%)	86 (15%)	76 (13%)
Newly diagnosed M1		690 (58%)	351 (59%)	347 (59%)	350 (59%)
Previously treated M0		33 (3%)	19 (3%)	13 (2%)	21 (4%)
Previously treated M1		34 (3%)	15 (3%)	15 (3%)	15 (3%)
**Gleason sum score**					
≤7		282 (24%)	122 (21%)	110 (19%)	117 (20%)
8–10		810 (68%)	421 (71%)	436 (74%)	425 (72%)
Unknown		92 (8%)	50 (8%)	46 (8%)	51 (9%)
**Aspirin or NSAID use**					
No		891 (75%)	448 (76%)	444 (75%)	445 (75%)
Yes		293 (25%)	145 (24%)	148 (25%)	148 (25%)
Planned or current hormone therapy*					
Orchidectomy		5 (0%)	4 (1%)	2 (0%)	3 (1%)
LHRH-based		1166 (98%)	581 (98%)	581 (98%)	582 (98%)
Bicalutamide		11 (1%)	7 (1%)	9 (2%)	8 (2%)
Maximum androgen blockade		2 (0%)	1 (0%)	0 (0%)	0 (0%)
**Time to starting hormone therapy (days)**					
Median (IQR)		−41 (−63 to −20)	−40 (−62 to −20)	−43 (−66 to −21)	−43 (−63 to −22)
Range		−105 to 77	−193 to 32	−108 to 45	−142 to 28
Missing		1	0	2	0
**Planned anti-androgen use**					
No		102 (9%)	67 (12%)	52 (9%)	57 (10%)
Short-term anti-androgen		876 (76%)	420 (72%)	437 (75%)	434 (75%)
Long-term anti-androgen		178 (15%)	95 (16%)	92 (16%)	91 (16%)
Missing		28	11	11	11
**Radiotherapy planned**					
No		844 (71%)	421 (71%)	424 (72%)	423 (71%)
Yes		340 (29%)	172 (29%)	168 (28%)	170 (29%)
**Does patient smoke?**					
No		1006 (87%)	492 (84%)	506 (86%)	496 (84%)
Yes		157 (13%)	93 (16%)	81 (14%)	92 (16%)
Missing on assessment		16	6	4	4
Assessment not received		5	2	1	1
**Does patient have diabetes**					
No		1058 (90%)	544 (92%)	535 (91%)	516 (88%)
Yes, type 1		29 (2%)	11 (2%)	26 (4%)	16 (3%)
Yes, type 2		89 (8%)	36 (6%)	30 (5%)	57 (10%)
Missing on assessment		3	0	0	3
Assessment not received		5	2	1	1
**Myocardial infarction**					
No		1146 (97%)	578 (98%)	575 (98%)	571 (97%)
Yes, but still fit for trial		31 (3%)	13 (2%)	14 (2%)	18 (3%)
Missing on assessment		2	0	2	3
Assessment not received		5	2	1	1
**Cerebrovascular disease**					
No		1164 (99%)	579 (98%)	583 (99%)	580 (98%)
Yes, but still fit for trial		13 (1%)	12 (2%)	6 (1%)	9 (2%)
Missing on assessment		2	0	2	3
Assessment not received		5	2	1	1
**Congestive heart failure**					
No		1172 (100%)	588 (99%)	588 (100%)	589 (100%)
Yes, but still fit for trial		5 (0%)	3 (1%)	2 (0%)	0 (0%)
Missing on assessment		2	0	1	3
Assessment not received		5	2	1	1
**Angina**					
No		1138 (97%)	567 (96%)	574 (97%)	569 (97%)
Yes, but still fit for trial		39 (3%)	24 (4%)	17 (3%)	20 (3%)
Missing on assessment		2	0	0	3
Assessment not received		5	2	1	1
**Hypertension**					
No		741 (63%)	384 (65%)	383 (65%)	385 (65%)
Yes, but still fit for trial		437 (37%)	206 (35%)	208 (35%)	204 (35%)
Missing on assessment		1	1	0	3
Assessment not received		5	2	1	1

Data are median (IQR), range, or n (%). NSAID=non-steroidal anti-inflammatory drug. LHRH=luteinising hormone-releasing hormone.

* Further information provided in the appendix.

**Table 2:** Treatment with zoledronic acid

		**Standard of care plus zoledronic acid (n=593)**	**Standard of care plus zoledronic acid and docetaxel (n=593)**
Numbers reporting starting		585 (99%)	544 (92%)
Numbers not reporting starting		8 (1%)	49 (8%)
Time to starting from randomisation, weeks		1·9 (1·0–2·9)	2·4 (1·6–3·7)
Time from starting hormone therapy to starting zoledronic acid, weeks		8·0 (5·0–11·3)	8·6 (5·9–11·9)
Time from starting to last administration, months		16·6 (7·8–23·2)	19·5 (9·1–23·4)
Reported reasons for stopping (if started):			
	Treatment complete	206 (35%)	218 (40%)
	Progressive disease	231 (39%)	119 (22%)
	Toxicity	43 (7%)	66 (12%)
	Unknown	38 (6%)	41 (8%)
	Treatment refusal*	26 (4%)	46 (8%)
	Dental treatment	11 (2%)	23 (4%)
	Death	15 (3%)	13 (2%)
	Intercurrent illness	5 (1%)	12 (2%)
	Other	10 (2%)	6 (1%)

* Including treatment refusal, patient decision, clinician decision, administrative reasons, and withdrawal from trial.

**Table 3:** Treatment with docetaxel

		**Standard of care plus docetaxel (n=592)**	**Standard of care plus zoledronic acid and docetaxel (n=593)**
Numbers reporting starting		546 (92%)	523 (88%)
Numbers not reporting starting		46 (8%)	70 (12%)
Time to starting from randomisation, weeks		2·1 (1·6–3·1)	2·4 (1·6–3·7)
Time from starting hormone therapy to starting docetaxel, weeks		8·6 (5·6–11·9)	8·7 (5·9–11·7)
Number of cycles reported:			
	0	46 (8%)	70 (12%)
	1	27 (5%)	22 (4%)
	2	17 (3%)	19 (3%)
	3	12 (2%)	18 (3%)
	4	13 (2%)	18 (3%)
	5	21 (4%)	24 (4%)
	6	456 (77%)	422 (71%)
Reported reasons for stopping (if started):			
	Treatment complete	454 (83%)	423 (81%)
	Toxicity	72 (13%)	66 (13%)
	Treatment refusal*	6 (1%)	8 (2%)
	Progressive disease	5 (1%)	8 (2%)
	Intercurrent illness	5 (1%)	7 (1%)
	Death	2 (0%)	5 (1%)
	Unknown	2 (0%)	5 (1%)
	Dental treatment	0 (0%)	1 (0%)

* Including treatment refusal, patient decision, clinician decision, and withdrawal from trial. Not all patients who reported stopping reason as “treatment complete” reported six cycles; similarly, not all patients reporting six cycles reported stopping reason as “treatment complete”.

**Table 4:** Treatments ever used at relapse, at the discretion of the treating clinician

		**Standard of care**	**Standard of care plus zoledronic acid**	**Standard of care plus docetaxel**	**Standard of care plus zoledronic acid and docetaxel**
Patients with progression		761	374	315	318
Reported new treatment		671 (88%)	303 (81%)	260 (83%)	257 (81%)
Reported (new) life-extending treatment		383 (50%)	172 (46%)	139 (44%)	136 (43%)
Life-extending treatment					
	Docetaxel	313 (41%)	136 (36%)	44 (14%)	49 (15%)
	Abiraterone	177 (23%)	72 (19%)	89 (28%)	88 (28%)
	Enzalutamide	66 (9%)	18 (5%)	25 (8%)	26 (8%)
	Cabazitaxel	26 (3%)	14 (4%)	22 (7%)	30 (9%)
	Radium-223	6 (1%)	1 (0%)	6 (2%)	3 (1%)
Other treatments					
	Anti-androgens	512 (67%)	234 (63%)	181 (57%)	174 (55%)
	Zoledronic acid	128 (17%)	50 (13%)	35 (11%)	36 (11%)
	Dexamethasone	104 (14%)	42 (11%)	39 (12%)	29 (9%)
	Diethylstilbestrol (also known as stilboestrol)	84 (11%)	43 (11%)	38 (12%)	41 (13%)
	Prednisolone	72 (9%)	22 (6%)	28 (9%)	23 (7%)
	Other chemotherapy*	26 (3%)	17 (5%)	21 (7%)	15 (5%)
	Other bisphosphonate†	22 (3%)	3 (1%)	8 (3%)	5 (2%)
	Strontium	12 (2%)	3 (1%)	2 (1%)	4 (1%)
	Cox-2 inhibition	0 (0%)	1 (0%)	0 (0%)	0 (0%)

* Not docetaxel or cabazitaxel.

† Not zoledronic acid

**Table 5:** Worst adverse event (grade) reported over entire time on trial

		**Standard of care (n=1184)**	**Standard of care plus zoledronic acid (n=593)**	**Standard of care plus docetaxel (n=592)**	**Standard of care plus zoledronic acid and docetaxel (n=593)**
**Safety population**					
Number of patients included in analysis*		1228	608	550	516
Grade 1–5 adverse event		1213 (99%)	604 (99%)	550 (100%)	515 (100%)
Grade 3–5 adverse event		399 (32%)	197 (32%)	288 (52%)	269 (52%)
Grade 5 adverse event		5	1	4	6
Most frequent adverse events reported as grade 3–5					
	Endocrine disorder (including impotence, hot flushes)	145 (12%)	74 (12%)	57 (10%)	64 (12%)
	Febrile neutropenia	15 (1%)	5 (<1%)	84 (15%)	74 (14%)
	Neutropenia (neutrophils)	6 (0%)	3 (<1%)	66 (12%)	62 (12%)
	General disorder (including lethargy, fever, asthenia)	46 (4%)	28 (5%)	34 (7%)	56 (11%)
	Musculoskeletal (including bone pain, generalised pain)	69 (6%)	35 (6%)	32 (6%)	44 (9%)
	Gastrointestinal disorder (including diarrhoea, abdominal pain, constipation, vomiting)	36 (3%)	19 (3%)	45 (8%)	37 (7%)
	Renal (including renal impairment, urinary-tract infection)	71 (6%)	30 (5%)	23 (4%)	25 (5%)
	Notable adverse events				
	Respiratory disorder (including dyspnoea, upper respiratory-tract infection)	27 (2%)	13 (2%)	29 (5%)	23 (4%)
	Cardiac disorder (including hypertension, myocardial infarction)	35 (3%)	19 (3%)	16 (3%)	19 (4%)
	Osteonecrosis of the jaw	0 (0%)	10 (2%)	0 (0%)	21 (4%)
	Nervous system other (including peripheral neuropathy)	20 (2%)	8 (1%)	19 (3%)	19 (4%)
	Nail changes	0 (0%)	0 (0%)	5 (1%)	4 (1%)
**ITT population**					
Number of patients included in analysis†		1173	587	579	563
Grade 1–5 adverse event		1160 (99%)	583 (99%)	577 (100%)	562 (100%)
Grade 3–5 adverse event		375 (32%)	184 (31%)	298 (51%)	296 (53%)
Grade 5 adverse event		4	1	4	7

Grade 5 adverse events were not necessarily treatment-related; similarly treatment-related deaths were not always grade 5 adverse events. ITT=intention-to-treat.

* Analysis by actual treatment initiated (irrespective of assigned study arm) in patients who underwent adverse event assessment.

† Analysis by assigned study arm in patients who underwent adverse event assessment.
