# Supplementary material for: Addition of docetaxel, zoledronic acid, or both to first-line long-term hormone therapy in prostate cancer (STAMPEDE): survival results from an adaptive, multiarm, multistage, platform randomised controlled trial
Source: Lancet. 2016 Mar 19;387(10024):1163–77. doi: 10.1016/S0140-6736(15)01037-5 (PMC4800035; doi:10.1016/S0140-6736(15)01037-5)
Supplement: Supplementary appendix [file mmc1.pdf]

THE LANCET

Supplementary appendix

This appendix formed part of the original submission and has been peer reviewed. We post it as supplied by the authors.

Supplement to: James ND, Sydes MR, Clarke NW, et al, for the STAMPEDE investigators. Addition of docetaxel, zoledronic acid, or both to first-line long-term hormone therapy in prostate cancer (STAMPEDE): survival results from an adaptive, multiarm, multistage, platform randomised controlled trial. *Lancet* 2015; published online Dec 21. http://dx.doi.org/10.1016/S0140-6736(15)01037-5.

SUPPLEMENT TO dx.doi.org/10.1016/S0140-6736(15)01037-5

Addition of docetaxel, zoledronic acid, or both to first-line long-term hormone therapy in prostate cancer (STAMPEDE): survival results from an adaptive, multi-arm, multi-stage, platform, randomised controlled trial

**Page**	**Section**
2	**Supplemental Figure 1**<br>Overall trial design
3	**Supplemental Figure 2**<br>Forest plots of treatment effect on failure-free survival within subgroups, by research comparison
5	**Supplemental Figure 3**<br>Overall survival for metastatic patients in the comparison SOC vs SOC+Doc
6	**Supplemental Figure 4**<br>Life-extending treatments used at relapse, at the discretion of the treating clinician, by arm
7	**Supplemental Figure 5**<br>Zoledronic acid at relapse, at the discretion of the treating clinician, by arm
8	**Supplemental Table 1**<br>Planned long-term hormone therapy by allocated treatment and metastases
9	**Supplemental Table 2**<br>Radiotherapy reporting according to nodal involvement, for non-metastatic patients, by arm
10	**Supplemental Table 3**<br>Breakdown of first FFS events by allocated treatment
11	**Investigator list**<br>Detailed investigator list (paper contains abridged version)
12	**Thanks**

Supplemental Figure 1: Overall trial design

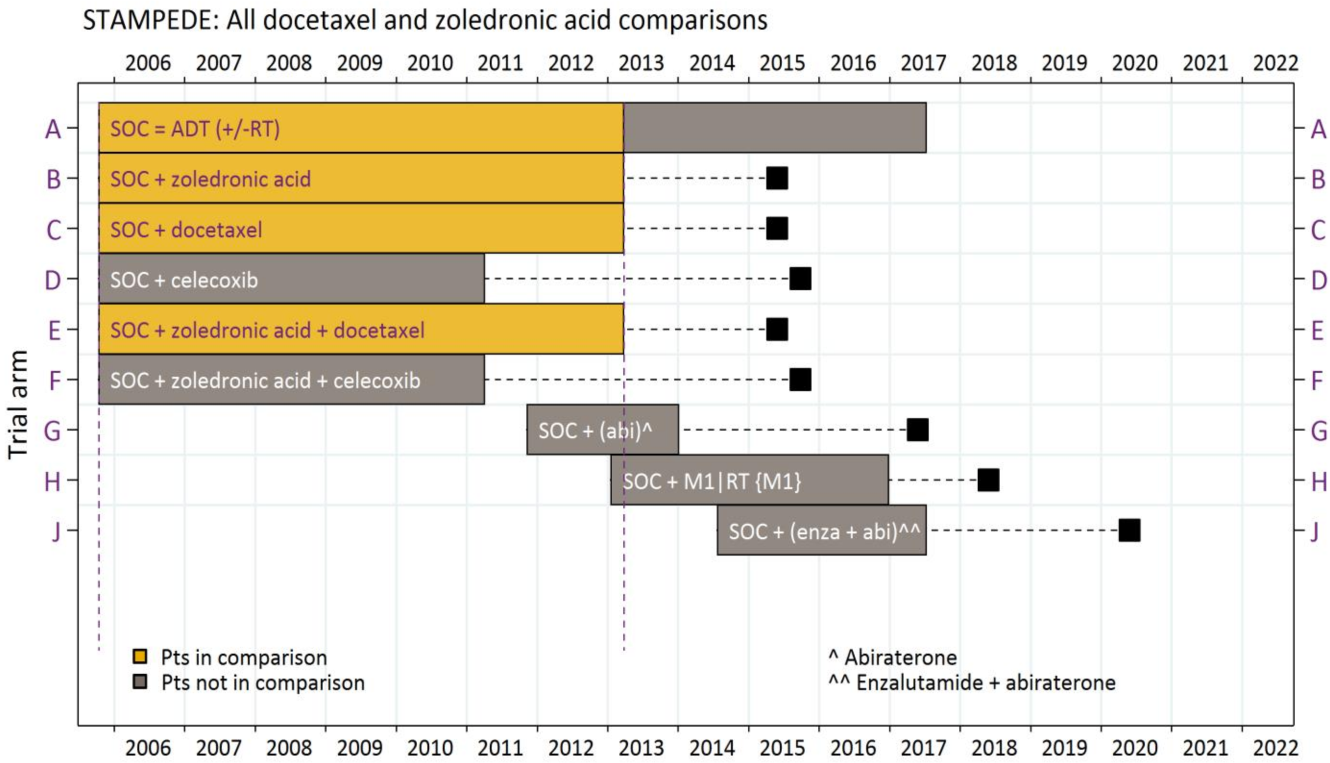

A = ~1200 pts --> ~404 primary outcome measure events
B = ~600 pts, C = ~600 pts, E = ~600 pts

Supplemental Figure 2: Forest plots of treatment effect on failure-free survival within subgroups, by research comparison

SOC vs SOC+ZA

Subgroup	SOC-only FFS/N	SOC+ZA FFS/N	Haz. Ratio (95% CI)
**Mets status**			
M0	176/460	88/227	1.00 (0.77, 1.29)
M1	585/724	286/366	0.89 (0.78, 1.03)
**Nodal status**			
N0	284/522	134/258	0.84 (0.68, 1.03)
N+	418/594	215/303	0.97 (0.82, 1.14)
NX	59/68	25/32	0.79 (0.48, 1.32)
**Gleason Sum Score (cats)**			
<=7	154/282	61/122	0.78 (0.58, 1.06)
8+	527/810	273/421	0.97 (0.84, 1.12)
unknown	80/92	40/50	0.72 (0.48, 1.09)
**Age at randomisation (cats)**			
Under 70	544/833	278/421	0.97 (0.84, 1.12)
70 or over	217/351	96/172	0.78 (0.61, 1.00)
**WHO PS 0 vs 1/2**			
0	561/922	288/465	1.02 (0.88, 1.17)
1+	200/262	86/128	0.69 (0.53, 0.89)
**NSAID/Aspirin use**			
No use	557/891	273/448	0.93 (0.80, 1.07)
Uses either	204/293	101/145	0.92 (0.72, 1.16)
**Is radiotherapy planned?**			
No RT planned	648/844	322/421	0.93 (0.82, 1.07)
RT planned	113/340	52/172	0.84 (0.60, 1.17)
**Previously treated**			
No	725/1117	356/559	0.91 (0.80, 1.04)
Yes	36/67	18/34	1.03 (0.57, 1.86)
Overall			0.92 (0.81, 1.04)

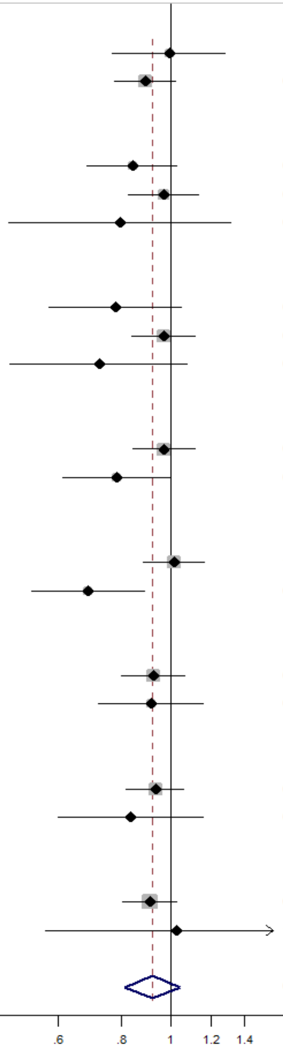

SOC vs SOC+Doc

Subgroup	SOC-only FFS/N	SOC+Doc FFS/N	Haz. Ratio (95% CI)
**Mets status**			
M0	176/460	63/230	0.60 (0.45, 0.80)
M1	585/724	252/362	0.61 (0.53, 0.71)
**Nodal status**			
N0	284/522	109/260	0.56 (0.44, 0.69)
N+	418/594	178/298	0.62 (0.52, 0.74)
NX	59/68	28/34	0.67 (0.41, 1.10)
**Gleason Sum Score (cats)**			
<=7	154/282	47/110	0.62 (0.45, 0.86)
8+	527/810	229/436	0.59 (0.50, 0.69)
unknown	80/92	39/46	0.62 (0.41, 0.94)
**Age at randomisation (cats)**			
Under 70	544/833	239/419	0.65 (0.56, 0.76)
70 or over	217/351	76/173	0.50 (0.38, 0.65)
**WHO PS 0 vs 1/2**			
0	561/922	233/461	0.62 (0.53, 0.72)
1+	200/262	82/131	0.57 (0.44, 0.74)
**NSAID/Aspirin use**			
No use	557/891	231/444	0.62 (0.53, 0.72)
Uses either	204/293	84/148	0.58 (0.45, 0.76)
**Is radiotherapy planned?**			
No RT planned	648/844	267/424	0.59 (0.52, 0.69)
RT planned	113/340	48/168	0.77 (0.55, 1.09)
**Previously treated**			
No	725/1117	301/564	0.60 (0.52, 0.68)
Yes	36/67	14/28	0.80 (0.42, 1.54)
Overall			0.61 (0.53, 0.70)

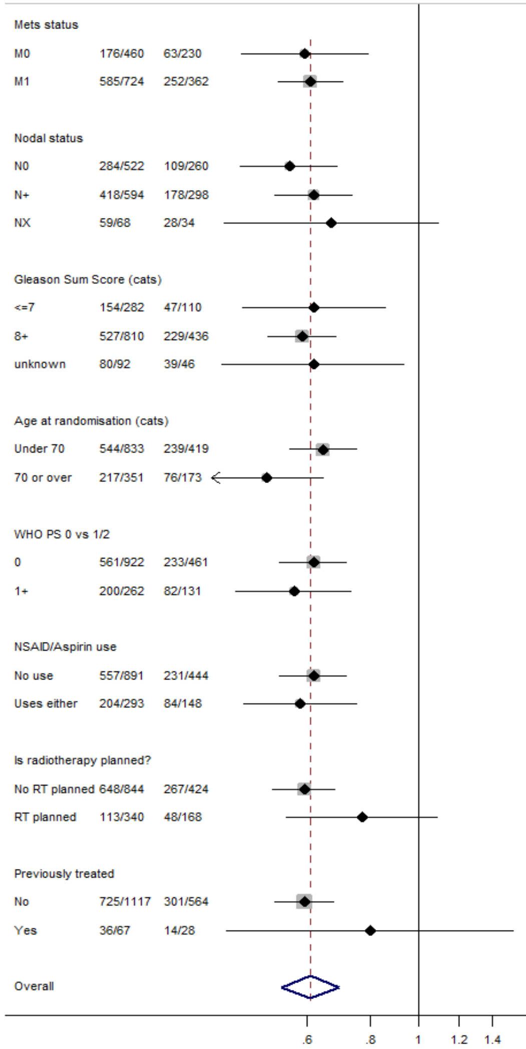

SOC vs SOC+ZA+Doc

Subgroup	SOC-only FFS/N	SOC+ZA+Doc FFS/N	Haz. Ratio (95% CI)
**Mets status**			
M0	176/460	63/228	0.68 (0.51, 0.91)
M1	585/724	255/365	0.60 (0.52, 0.70)
**Nodal status**			
N0	284/522	123/265	0.68 (0.54, 0.84)
N+	418/594	166/293	0.58 (0.48, 0.69)
NX	59/68	29/35	0.56 (0.34, 0.92)
**Gleason Sum Score (cats)**			
<=7	154/282	53/117	0.59 (0.43, 0.82)
8+	527/810	220/425	0.59 (0.50, 0.69)
unknown	80/92	45/51	0.65 (0.44, 0.96)
**Age at randomisation (cats)**			
Under 70	544/833	235/416	0.65 (0.56, 0.76)
70 or over	217/351	83/177	0.51 (0.40, 0.67)
**WHO PS 0 vs 1/2**			
0	561/922	231/461	0.65 (0.56, 0.76)
1+	200/262	87/132	0.50 (0.39, 0.65)
**NSAID/Aspirin use**			
No use	557/891	239/445	0.64 (0.55, 0.74)
Uses either	204/293	79/148	0.55 (0.42, 0.72)
**Is radiotherapy planned?**			
No RT planned	648/844	276/423	0.61 (0.53, 0.70)
RT planned	113/340	42/170	0.69 (0.48, 0.99)
**Previously treated**			
No	725/1117	302/557	0.60 (0.52, 0.68)
Yes	36/67	16/36	0.84 (0.44, 1.60)
Overall			0.62 (0.54, 0.70)

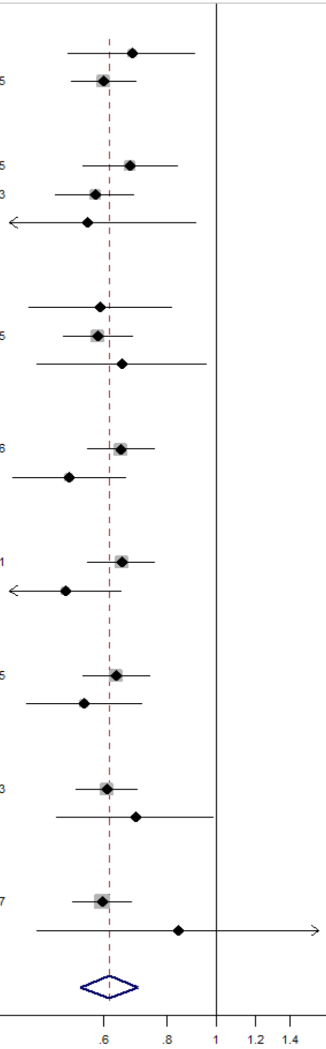

N.B.All p-values were statistically non-significant: AvsB all $p>0\cdot09$ except WHO PS $p=0\cdot008$, PSA $p=0\cdot093$, time-period $p=1\cdot000$; AvsC all $p>0\cdot15$, PSA $p=0\cdot886$, time-period $p=1\cdot000$; AvsE all $p>0\cdot14$, PSA $p=0\cdot249$, time-period $p=1\cdot000$.

Supplemental Figure 3: Overall survival for metastatic patients in the comparison SOC vs SOC+Doc

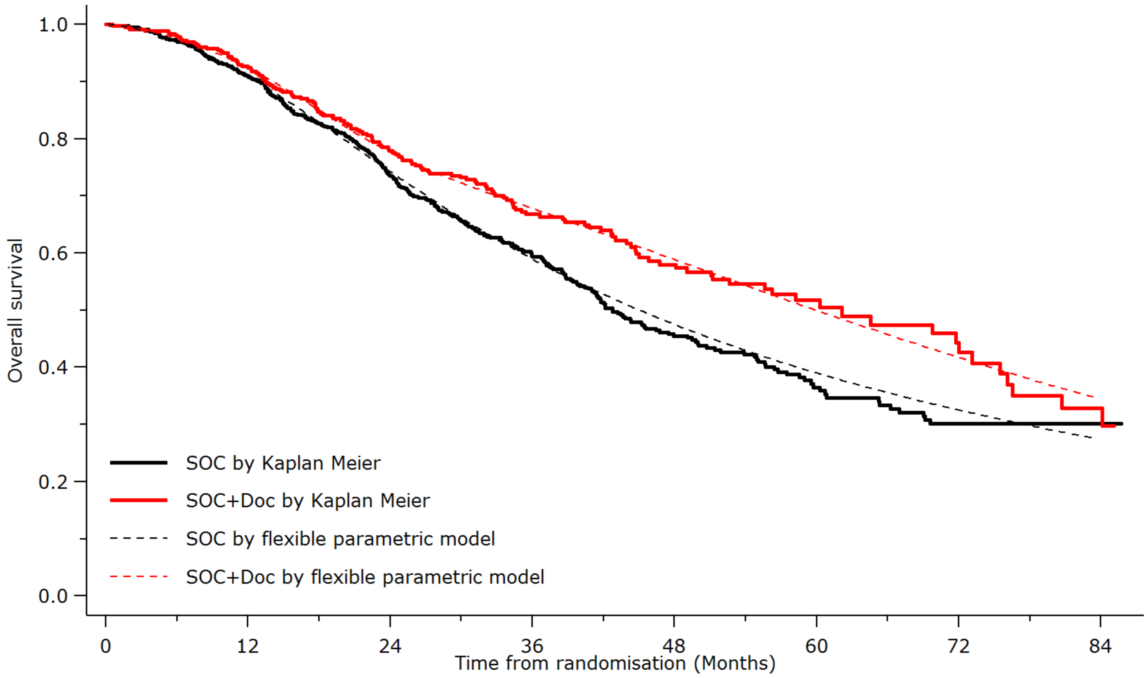

Number of patients (events)														
SOC	724	(65)	646	(121)	474	(78)	262	(53)	137	(21)	60	(10)	26	(0)	13
SOC+Doc	362	(27)	326	(50)	250	(30)	155	(16)	91	(8)	38	(6)	25	(5)	11

Supplemental Figure 4: Life-extending treatments used at relapse, at the discretion of the treating clinician, by arm

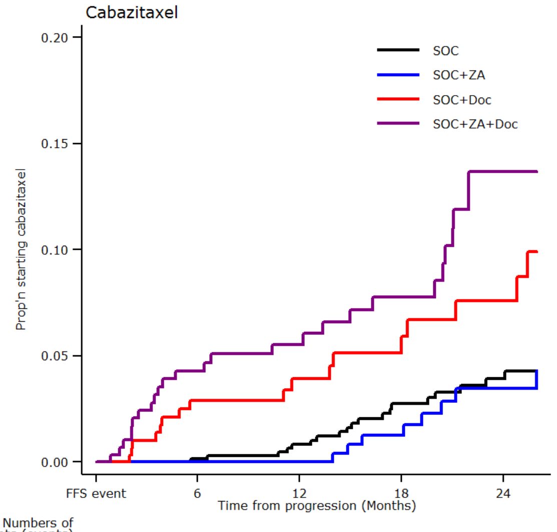

Numbers of patients (events)

	FFS event		6		12		18		24
SOC	761	(1)	661	(4)	538	(9)	392	(4)	265
SOC+ZA	374	(0)	326	(0)	268	(3)	197	(4)	130
SOC+Doc	315	(8)	245	(2)	184	(2)	122	(3)	92
SOC+ZA+Doc	318	(12)	246	(3)	188	(4)	135	(7)	82

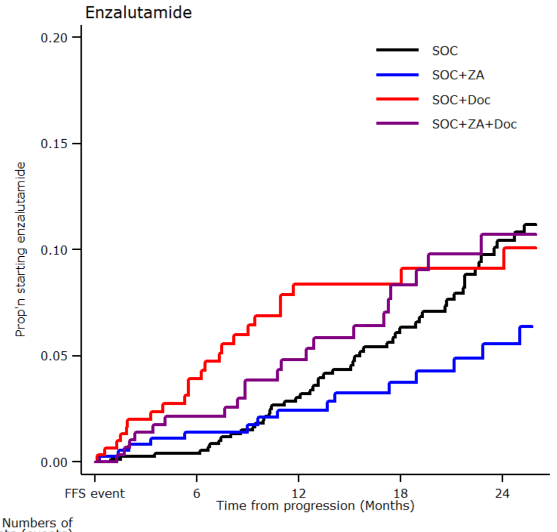

Numbers of patients (events)

	FFS event		6		12		18		24
SOC	761	(3)	660	(16)	531	(16)	391	(14)	256
SOC+ZA	374	(5)	321	(3)	261	(3)	193	(3)	126
SOC+Doc	315	(11)	242	(10)	178	(0)	126	(1)	95
SOC+ZA+Doc	318	(6)	250	(6)	188	(6)	138	(3)	88

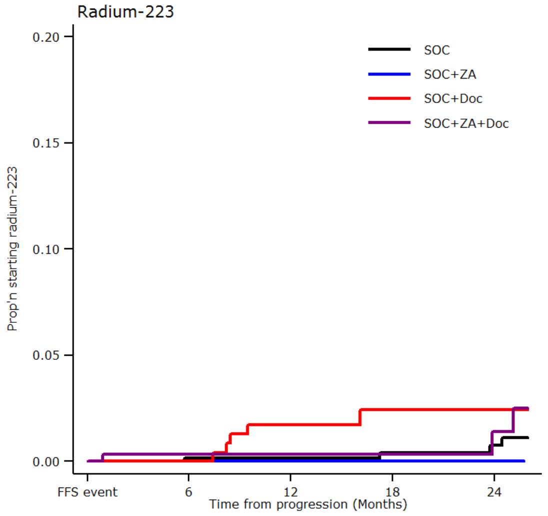

Numbers of patients (events)

	FFS event		6		12		18		24
SOC	761	(1)	661	(0)	542	(1)	401	(1)	271
SOC+ZA	374	(0)	326	(0)	268	(0)	200	(0)	132
SOC+Doc	315	(0)	252	(4)	187	(1)	125	(0)	93
SOC+ZA+Doc	318	(1)	253	(0)	196	(0)	146	(1)	94

Supplemental Figure 5: Zoledronic acid at relapse, at the discretion of the treating clinician, by arm

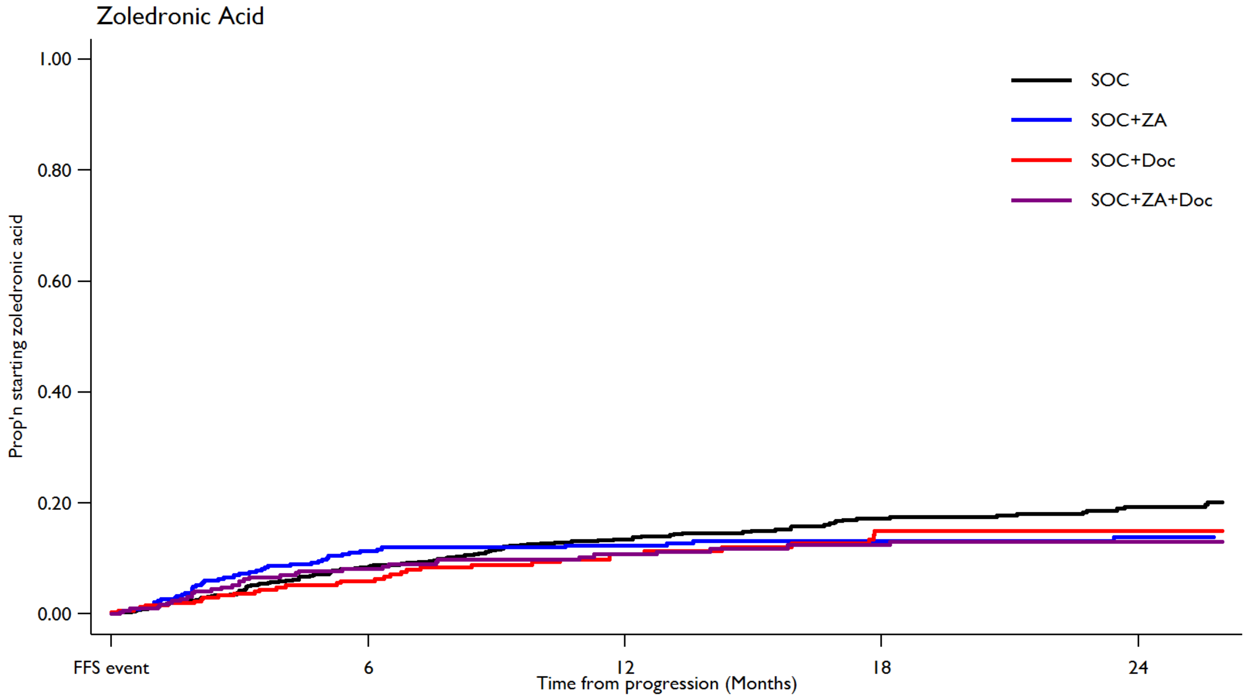

Numbers of patients (events)									
SOC	761	(60)	611	(32)	476	(18)	347	(7)	234
SOC+ZA	374	(40)	289	(3)	234	(2)	174	(1)	114
SOC+Doc	315	(17)	236	(11)	169	(6)	109	(0)	82
SOC+ZA+Doc	318	(23)	234	(6)	178	(3)	130	(1)	85

Supplemental Table 1: Planned long-term hormone therapy by allocated treatment and metastases

	SOC (n=1184)		SOC+ZA (n=593)		SOC+Doc (n=592)		SOC+ZA+Doc (n=593)		
**Planned long-term HT**	**M0**	**M1**	**M0**	**M1**	**M0**	**M1**	**M0**	**M1**	**Total**
LHRH agonist	102	157	58	71	59	77	59	73	656
LHRH antagonist	42	70	13	44	15	38	11	45	278
LHRH (unspecified)	307	488	146	249	147	245	149	245	1976
Orchidectomy	0	5	2	2	0	2	1	2	14
Maximum androgen blockade	0	2	1	0	0	0	0	0	3
Bicalutamide	9	2	7	0	9	0	8	0	35
**Total**	**460**	**724**	**227**	**366**	**230**	**362**	**228**	**365**	**2962**

Supplemental Table 2: Radiotherapy reporting according to nodal involvement, for non-metastatic patients, by arm

	SOC	SOC+ZA	SOC+Doc	SOC+ZA+Doc
**Node-negative patients**				
Radiotherapy reported	198 (71%)	94 (73%)	101 (71%)	90 (61%)
Radiotherapy not reported	82 (29%)	34 (27%)	41 (29%)	57 (39%)
**Node-positive patients**				
Radiotherapy reported	91 (51%)	42 (44%)	30 (34%)	40 (49%)
Radiotherapy not reported	87 (49%)	53 (56%)	57 (66%)	41 (51%)

Supplemental Table 3: Breakdown of first FFS events by allocated treatment

**First Reported Progression**	**SOC**		**SOC+ZA**		**SOC+Doc**		**SOC+ZA+Doc**	
Patients with progression	*761*		*374*		*315*		*318*	
PCa-related death	9	1%	8	2%	11	3%	17	5%
Distant metastases	88	12%	37	10%	38	12%	40	13%
Lymph node involvement	15	2%	2	1%	3	1%	5	2%
SRE	3	0.4%	0	0%	1	0.3%	0	0%
Local progression	17	2%	10	3%	12	4%	12	4%
PSA failure only	629	83%	317	85%	250	79%	244	77%

*Note: If two events are reported at the same time "worst event" is reported in this table; the table is ordered by this "worst event".

STAMPEDE Investigator List

Key: Site (N1/N2: Site Principle Investigator; Other randomising consultants) where N1 = accrual from trial start to end of recruitment to docetaxel and zoledronic acid arms (Oct-2005 to Mar-2013) and N2 = accrual from trial start to data freeze (Oct-2005 to 13-May-2015)

UNITED KINGDOM

Aberystwyth, Bronglais General Hospital (3/4: Sajid Durrani)
Ashford, William Harvey Hospital (4/11: Carys Thomas; Natasha Mithal)
Aylesbury, High Wycombe & Stoke Mandeville Hospital (6/12: Ami Sabharwal; Philip Camilleri, Christopher Alcock, Andrew Protheroe, Joanne Brady)
Ayr, Ayr Hospital (27/45: Hilary Glen; Jawaher Ansari, Rana Mahmood)
Barnet, Barnet General Hospital (8/17: Ursula McGovern; Andrew Eichholz)
Barnstaple, North Devon District Hospital (15/25: Denise Sheehan)
Basingstoke, Basingstoke and North Hampshire Hospital (11/17: Richard Shaffer; Teresa Guerrero-Urbano)
Bath, Royal United Hospital (23/53: Olivera Frim; Mark Beresford, Hugh Newman, Penny Kehagioglou)
Belfast, Belfast City Hospital (97/170: Joe O'Sullivan; Darren Mitchell, Poh Lin Shum, David Stewart, Suneil Jain)
Birmingham, Birmingham Heartlands Hospital (12/32: Anjali Zarkar)
Birmingham, City Hospital (11/24: Daniel Ford)
Birmingham, Queen Elizabeth Hospital (127/172: Nicholas James; Emilio Porfiri, Daniel Ford)
Blackburn, Royal Blackburn Hospital (32/61: Omi Parikh)
Bolton, Royal Bolton Hospital (8/24: Tony Elliott; Michael Pantelides)
Boston, Pilgrim Hospital (4/19: Thiagarajan Sreenivasan; Miguel Panades)
Bournemouth, Royal Bournemouth Hospital (52/84: Sue Brock; Joe Davies)
Bradford, Bradford Royal Infirmary (9/24: Simon Brown)
Brighton, Royal Sussex County Hospital (50/81: Angus Robinson; George Plataniotis, David Bloomfield, Marie Wilkins)
Bristol, Bristol Haematology & Oncology Centre (34/78: Amit Bahl; Mark Beresford, Paula Wilson, Serena Hilman, Chris Herbert)
Burnley, Burnley General Hospital (37/69: Natalie Charnley; Omi Parikh)
Burton-on-Trent, Queens Hospital (43/79: Shan Chetiyawardana; Dakshinamoorthy Muthukumar, Mike Smith-Howell, Pugazhenthi Pattu, Prabir Chakraborti)
Bury St Edmunds, West Suffolk Hospital (7/17: Cathryn Woodward; Yvonne Rimmer)
Cambridge, Addenbrooke's Hospital (0/4: Danish Mazhar)
Canterbury, Kent and Canterbury Hospital (33/61: Carys Thomas; Natasha Mithal, Rakesh Raman, Albert Edwards)
Cardiff, Velindre Hospital (226/314: Malcolm Mason; Jim Barber, Jason Lester, John Staffurth, Jacob Tanguay, Nachiappan Palaniappan, Satish Kumar, Michael Button, Diana Mort)
Carlisle, Cumberland Infirmary (6/9: Anil Kumar; Norma Sidek)
Chelmsford, Broomfield Hospital (32/61: Abdel Hamid; Udaiveer Panwar, Priscilla Leone)
Cheltenham, Cheltenham General Hospital (2/17: Jo Bowen; Peter Jenkins)
Chester, Countess of Chester Hospital (27/66: Azman Ibrahim)
Colchester, Essex County Hospital (2/34: Bruce Sizer; Muthar Kumar)
Coventry, University Hospital Coventry and Warwickshire (0/30: Jane Worlding; Andrew Stockdale)
Crewe, Leighton Hospital (15/41: James Wylie)
Darlington, Darlington Memorial Hospital (17/34: Mohammed Kagzi; John Hardman, Clive Peedell, Tanmay Mukhopadhyay)
Derby, Royal Derby Hospital (52/100: Prabir Chakraborti; Dakshinamoorthy Muthukumar, Pugazhenthi Pattu)
Doncaster, Doncaster Royal Infirmary (5/22: Mymoona Alzouebi; Catherine Ferguson)
Dorchester, Dorset County Hospital (19/24: Perric Crellin; Stephen Andrews)
Dudley, Russells Hall Hospital (46/65: Pek Keng-Koh; Prakash Ramachandra)
Durham, University Hospital North Durham (17/17: Rhona McMenemin)
Eastbourne, Eastbourne District General Hospital (32/52: Fiona McKinna)
Edinburgh, Western General Hospital (71/105: Duncan McLaren)

Exeter, Royal Devon and Exeter Hospital (102/152: Denise Sheehan; Rajaguru Srinivasan, Victoria Ford)
Gillingham, Medway Maritime Hospital (9/18: Henry Taylor)
Glasgow, Beatson West of Scotland Cancer Centre (223/291: Rob Jones; Martin Russell, Jan Wallace, John Graham, Rana Mahmood, Carolynn Lamb, Abdulla Al-hasso, Balaji Venugopal)
Guildford, Royal Surrey County Hospital (71/106: Robert Laing; Julian Money-Kyrle, Sara Khaksar, Katie Wood, Teresa Guerrero-Urbano)
Harlow, Princess Alexandra Hospital (20/34: Nishi Gupta; Lucinda Melcher)
Hereford, Hereford County Hospital (35/53: Warren Grant; Audrey Cook)
High Wycombe, Wycombe Hospital (28/45: Ami Sabharwal; Andrew Protheroe, Philip Camilleri, Thinn Pwint, Gerard Andrade)
Huddersfield, Huddersfield Royal Infirmary (48/76: Uschi Hofmann)
Hull, Castle Hill Hospital (75/100: Matthew Simms; John Hetherington)
Inverness, Raigmore Hospital (43/72: Neil McPhail; Azmat Sadozye, Kay Kelly, Carol Macgregor)
Ipswich, Ipswich Hospital (42/84: Robert Brierly; Ramachandran Venkitaraman, Christopher Scrase, Gautam Banerjee)
Keighley, Airedale General Hospital (18/39: Simon Brown; Michael Crawford, Clara Sentamans)
Kidderminster, Kidderminster General Hospital (10/23: Mark Churn; Lisa Capaldi)
Larbert, Forth Valley Royal Hospital (3/22: Norma Sidek)
Leeds, St James University Hospital (Leeds) (16/54: William Cross; Stephen Prescott, David Bottomley, Alan Paul, Carmel Loughrey, Sunjay Jain, Ann Henry, Peter Whelan)
Lincoln, Lincoln County Hospital (6/32: Thiagarajan Sreenivasan; David Ballesteros-Quintail, Miguel Panades, Karin Baria)
Liverpool, Royal Liverpool University Hospital (41/65: Zafar Malik; Chinnamani Eswar, Peter Robson)
Liverpool, University Hospital Aintree (7/16: Peter Robson)
London, Charing Cross Hospital (1/25: Alison Falconer)
London, Guy's Hospital (83/138: Simon Chowdhury; Peter Harper, Stephen Morris, Rick Popert, Ronald Beaney)
London, Hammersmith Hospital (3/4: Alison Falconer; Stephen Mangar)
London, North Middlesex Hospital (9/21: Jackie Newby; Anna Thompson, Farhad Neave, Stephen Karp)
London, Queen Elizabeth Hospital (12/18: Simon Hughes)
London, Royal Free Hospital (20/34: Maria Vilarino-Varela; Katherine Pigott)
London, Royal Marsden Hospital (6/9: Vincent Khoo)
London, St Bartholomews Hospital (0/6: Karen Tipples; Paula Wells)
London, St George's Hospital (9/29: Lisa Pickering)
London, St Mary's Hospital (5/5: Alison Falconer; Simon Stewart)
London, University College Hospital (21/38: Ursula McGovern; Stephen Harland, Heather Payne)
Maidstone, Maidstone Hospital (54/86: Sharon Beesley; Amanda Clarke, Henry Taylor)
Manchester, Christie Hospital (96/142: Noel Clarke; Tony Elliott, James Wylie, Jacqueline Livsey, John Logue, Richard Cowan, Ananya Choudhurey)
Manchester, Withington Hospital (7/7: Vijay Sangar)
Margate, Queen Elizabeth The Queen Mother Hospital (4/15: Carys Thomas; Rakesh Raman, Natasha Mithal)
Middlesbrough, James Cook University Hospital (61/90: Clive Peedell; John Hardman, Hans Van der Voet, Devadasan Shakespeare, David Chadwick)
Newcastle-upon-Tyne, Freeman Hospital (0/56: Ashraf Azzabi; Rhona McMenemin, John Frew)
Northwood, Mount Vernon Hospital (58/98: Peter Hoskin; Roberto Alonzi, Peter Ostler, Nicola Anyamene, Robert Hughes, Jeanette Dickson, Charlotte Westbury)
Nottingham, Nottingham University Hospitals, City Campus (61/97: Santhanam Sundar; Jamie Mills, Eliot Chadwick)
Nuneaton, George Eliot Hospital (0/9: Andrew Chan)
Oldham, Royal Oldham Hospital (10/45: Jacqueline Livsey; Ananya Choudhury)
Oxford, Churchill Hospital (106/140: Andrew Protheroe; David J Cole)
Poole, Poole Hospital (22/52: Sue Brock; Joseph Davies, Joe Davies)
Portsmouth, Queen Alexandra Hospital (58/137: Joanna Gale)
Preston, Royal Preston Hospital (100/179: Alison Birtle; Omi Parikh, Marcus Wise)
Reading, Royal Berkshire Hospital (18/34: Paul Rogers; Helen O'Donnell, Richard B Brown)
Redditch, Alexandra Hospital (0/12: Joanna Hamilton)
Romford, Queen's Hospital (65/101: Stephanie Gibbs; Ramachandran Subramaniam)

Salford, Salford Royal Hospital (32/52: Noel Clarke; Maurice Lau, Tony Elliott, Anna Tran, Satish Maddineni)
Scarborough, Scarborough General Hospital (14/59: Mohan Hingorani)
Sheffield, Weston Park Hospital (68/99: Catherine Ferguson; Peter Kirkbride, Mymoona Alzouebi, Tathagata Das)
Shrewsbury, Royal Shrewsbury Hospital (76/132: Narayanan Srihari; Ravi Prashant)
South Shields, South Tyneside District Hospital (2/4: Ashraf Azzabi)
Southampton, Southampton General Hospital (32/66: Catherine Heath; Simon Crabb, Matthew Wheater)
Southend, Southend University Hospital (76/102: David Tsang; Imtiaz Ahmed, Olivia Chan, Naveed Sarwar)
Southport, Southport and Formby District General Hospital (15/37: Neeraj Bhalla; Chinnamani Eswar, Asha Sivapalasuntharam)
St Leonards-on-Sea, Conquest Hospital (25/31: Fiona McKinna; Kathryn Lees, Sharon Beesley)
Stevenage, Lister Hospital (19/27: Robert Hughes)
Stockport, Stepping Hill Hospital (58/88: John Logue; Adebanji Adeyoju)
Stockton-on-Tees, University Hospital of North Tees (2/10: Devadasan Shakespeare)
Stoke-on-Trent, Royal Stoke Hospital (22/57: Fawzi Adab; Rajanee Bhana)
Sunderland, Sunderland Royal Hospital (29/34: Ashraf Azzabi; Ian Pedley)
Sutton Coldfield, Good Hope Hospital (3/14: Daniel Ford)
Sutton, Royal Marsden Hospital (73/126: David Dearnaley; Chris Parker, Robert Huddart, Vincent Khoo)
Sutton-in-Ashfield, King's Mill Hospital (23/36: Daniel Saunders; Georgina Walker)
Swansea, Singleton Hospital (115/160: John Wagstaff; Gianfilippo Bertelli, Delia Pudney, Mau-Don Phan)
Swindon, Great Western Hospital (21/41: Omar Khan; David J Cole, Esme Hill)
Taunton, Musgrove Park Hospital (65/103: Emma Gray; John Graham, Mohini Varughese, Manjusha Keni, George Plataniotis)
Torquay, Torbay District General Hospital (68/114: Anna Lydon; Rajaguru Srinivasan)
Warrington, Warrington Hospital (54/93: Isabel Syndikus; Shaun Tolan)
Warwick, Warwick Hospital (0/13: Andrew Stockdale)
Weston Super Mare, Weston General Hospital (6/12: Serena Hilman)
Whitehaven, West Cumberland Hospital (1/1: Anil Kumar; Jonathon Nicoll)
Wigan, Royal Albert Edward Infirmary (13/25: Anna Tran; Richard Cowan)
Wirral, Clatterbridge Centre for Oncology (57/107: Shaun Tolan; John Littler, Isabel Syndikus, Amir Montazeri, Azman Ibrahim)
Wolverhampton, New Cross Hospital (0/19: Ian Sayers)
Worcester, Worcestershire Royal Hospital (24/44: Lisa Capaldi; Jo Bowen)
Worthing, Worthing Hospital (51/75: Ashok Nikapota; David Bloomfield, Fiona Castell)
Yeovil, Yeovil District Hospital (0/4: Geoffrey Sparrow; Emma Gray)

SWITZERLAND

Aarau, Hirslanden Medical Centre (3/3: Razvan Popescu)
Basel, Universitätsspital Basel (1/3: Cyrill Rentsch; Bettina Seifest)
Berne, Inselspital (University Hospital Berne) (1/3: George Thalmann; Beat Roth)
Chur, Kantonsspital Graubünden (17/24: Raeto Strebel; Richard Cathomas)
Lausanne, Centre Hospitalier Universitaire Vaudois (CHUV) (3/5: Dominik Berthold; Patrice Jichlinski, Fernanda Herrera)
St Gallen, Kantonsspital St Gallen (5/8: Daniel Engeler; Stefan Prensser)
Zurich, Triemlispital (1/1: Donat Durr; Daniele Siciliano)

Thanks

We recognise the efforts of all of the staff at sites who have been fundamental in the conduct of the trial and thank them.

We also recognise all of the patients who have chosen to join the trial, and their families and friends who supported them. Our thanks to them all.
